# Zapałowicz’s *Conspectus florae Galiciae criticus*: Clarification of publication dates for nomenclatural purposes and bibliographic notes

**DOI:** 10.3897/phytokeys.155.51072

**Published:** 2020-08-07

**Authors:** Beata Paszko, Agnieszka Nikel, Aldona Mueller-Bieniek, Wojciech Paul

**Affiliations:** 1 W. Szafer Institute of Botany, Polish Academy of Sciences, Lubicz 46, PL-31-512, Kraków, Poland Polish Academy of Sciences Kraków Poland

**Keywords:** bibliography, botanical nomenclature, history of publication, Hugo Zapałowicz, priority, taxonomic botanical literature, verification

## Abstract

Work on the catalogue of type specimens of vascular plants deposited in the KRAM herbarium has highlighted uncertainties and errors in references to place of valid publication of numerous taxa described by Hugo Zapałowicz in his *Conspectus florae Galiciae criticus* – *Krytyczny przegląd roślinności Galicyi* (1904–1914). Zapałowicz published his work in an excerpt series, a serial publication and a multi-volume book, with much duplication amongst these three different forms. Despite the importance of this work, no studies have clarified the dates of publication of its various parts, as relevant to the nomenclature of numerous new taxa of Central European vascular plants described therein: 94 species and hybrids, 10 subspecies and more than 2000 other infraspecific taxa. Here, the publication dates of the component parts of Zapałowicz’s work are clarified and discussed. Archival sources that made it possible to determine publication dates of these works are described in detail.

## Introduction

**Figure 1.**
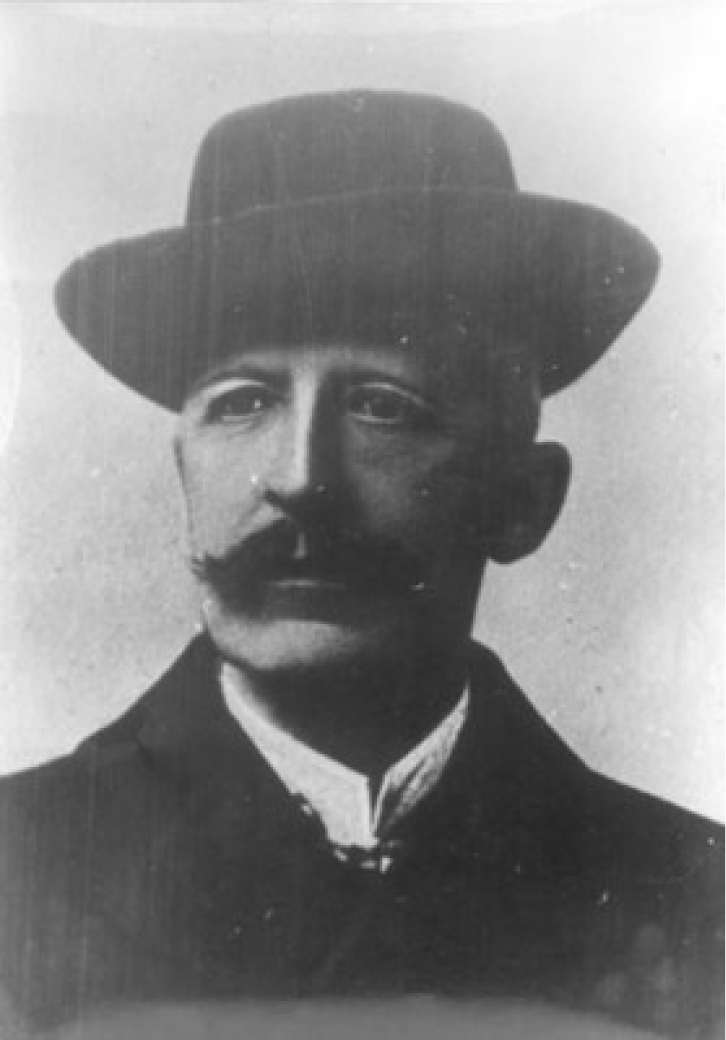
Hugo Zapałowicz (1852–1919). Image from http://www.cotg.pttk.pl/newsy/img/zapalowicz.jpg [Accessed September 2019].

An eminent Polish naturalist, Hugo Zapałowicz (Fig. 1), was born on 15 November 1852 in Laibach (now Ljubljana in Slovenia) and died 20 November 1917 in Perovsk (now Kyzylorda in Kazakhstan). After graduating from high school, Zapałowicz studied law and in 1876 was awarded a doctorate at the Faculty of Law, Jagiellonian University, Kraków. Following this, Zapałowicz worked as a military lawyer, but botany, taken up early in his life, was his major passion. In 1894, Zapałowicz became a Member of the Academy of Arts and Sciences in Kraków (AAS, since 1920: Polish AAS – PAAS; in Polish, respectively: Akademia Umiejętności – AU, Polska Akademia Umiejętności – PAU) and, for a long time, had been co-operating with the Museum of the Physiographic Commission of the AAS, studying its abundant herbarium. Zapałowicz retired in 1905, after a 35 years law career, but returned to the military during World War I. Imprisoned by Russian troops, he died as a prisoner of war. Throughout his life, Zapałowicz devoted all of his leisure time to investigating the flora of the Carpathian Mountains and Galicia (today mostly the territory of western Ukraine and south-eastern Poland, Eastern Europe) (Rouppert 1918; Hryniewiecki 1953; Zdebska 1978–1979; Zdebski 1978–1979; Majkowska 2006).

In 1904, Zapałowicz began publishing his most distinguished work in the field of botany, titled *Conspectus florae Galiciae criticus* – *Krytyczny przegląd roślinności Galicyi* (1904−1914) [= A critical review of the flora of Galicia] (hereafter, the *Conspectus*). The *Conspectus* was a multipart work that appeared over 11 years and was published in three forms: as an excerpt series, a serial publication and a multi-volume book. The excerpts were written in French, whereas the serial and books were written in Polish; in all three forms, Latin was used for species description and taxonomic remarks. Although unfinished, the *Conspectus* is a monumental work that provided the first (but incomplete) enumeration of the vascular plants (1222 species in 52 families, including addenda) of Galicia, including descriptions of new taxa (49 species, 50 hybrids, 10 subspecies and more than 2000 other infraspecific taxa). Entries in the *Conspectus* include accepted names, selected synonyms (basionyms where appropriate), detailed Latin descriptions of most taxa, taxonomic notes, localities of herbarium specimens studied and regional distributions; taxonomic keys were not included. The *Conspectus* was issued as a book in three volumes; a projected 4^th^ volume was never published. Volume 1 covers pteridophytes, monocotyledons and gymnosperms (Polypodiaceae-Coniferae) and Volumes 2 and 3 cover dicotyledons, including families from Betulaceae to Caryophyllaceae. Content that would have appeared in Volume 4 was published only in serial form, covering dicotyledon families from Papaveraceae to Violaceae. The work was based almost entirely on the herbarium collection housed at the Museum of the Physiographic Commission of the AAS in Kraków, including the author’s own collection (Zapałowicz 1904, 1906b), currently housed at the KRAM herbarium (with some duplicates at KRA).

We identified 65 different publication events related to the *Conspectus*, including numerous duplicate nomenclaturally-redundant publications. These events comprise a 30 part excerpt series (representing 32 publication events), a 30 part serial publication and a three volume book. All but one of them were published under the same Polish title. Stafleu and Cowan (1988) listed the *Conspectus* (no. 18.599) under its alternative Latin title, even though the Latin title was included in only 33 of the publication events (for details see Appendix I).

The 30 individual parts that make up the *Conspectus* were published separately in a Polish journal, *Rozprawy Wydziału Matematyczno-Przyrodniczego Akademii Umiejętności*, *Dział B. Nauki Biologiczne* (*Seria 3*) (hereafter, the *Rozprawy*). The content of the first 21 parts of the *Rozprawy* was brought together and re-published in a three volume book, along with Addenda, Corrigenda and Index generum sections at the end of each volume. The 30 excerpts (selections) were published in advance of the serial publications and the books in an international monthly journal, *Bulletin International de l’Académie des Sciences de Cracovie*: *Classe des Sciences Mathématiques et Naturelles* (Vols. 1904–1909) and its successor, *Bulletin International de l’Académie des Sciences de Cracovie*: *Classe des Sciences Mathématiques et Naturelles*; *Série B. Sciences Naturelles* (Vols. 1910–1914) (hereafter, the *Bulletin*). Parts 1 to 21 of the *Conspectus* were published in the *Rozprawy* and in book form and excerpts from them were published in the *Bulletin*. Parts 22 to 30 were published in the *Rozprawy* and excerpts from them were published in the *Bulletin*.

Zapałowicz’s names in the *Conspectus* were usually published twice and sometimes even three times, but none of these duplicate or triplicate publications indicated that the name had already been published elsewhere. A few of Zapałowicz’s names, for example, Carex × bogdanensis, C. × paczoskii and C. × raciborskii, were published exclusively in the Addenda sections at the end of book volumes. Zapałowicz’s names of new taxa, published in each of the various forms of the *Conspectus*, meet the conditions of the International Code of Nomenclature (ICN) for valid and effective publication (Art. 29, 30 of ICN; Turland et al. 2018). Zapałowicz’s new names also meet the conditions for valid publication (Art. 32 of ICN; Turland et al. 2018); all are accompanied by Latin (rarely also French, exceptionally Polish) descriptions and/or diagnoses. Due to the multiple and mostly redundant instances of publication of Zapałowicz’s work, however, there is chaos in the citations of the correct place of publication of his names in the taxonomic literature, including in Stafleu and Cowan (1988), authoritative online botanical nomenclature databases of taxon names and nomenclatural acts (International Plant Names Index (IPNI), Tropicos (Missouri Botanical Garden 2019)), as well as in the primary literature (Mitka and Starmühler 2000; Optasyuk and Shevera 2011; Ziman et al. 2015; Wacławska-Ćwiertnia and Mitka 2016; Barberá et al. 2018). Stafleu and Cowan (1988) assessed the status of Zapałowicz’s book volumes as “Reprinted and to be cited from Rozpr. Akad. Umiej. Krakow ser. B”. However, based on dates of publication of volumes of the *Rozprawy* that included papers presented at the AAS meetings in 1906, 1908 and 1911, we concluded that, in several cases, material published in book form could have appeared simultaneously or prior to that published in the journal. IPNI (2019) usually variously cites the *Bulletin* or book form of the *Conspectus* as the correct place of publication of Zapałowicz’s names; rarely IPNI cites both sources (e.g. for “*Silene berdaui* Zapał., Bull. Acad. Cracovie 1911. B, 286; Consp. Fl. Galic. Crit. iii. 182 (1911)”) or even a secondary source is cited as a second place of publication (e.g. for “*Thlaspi tatrense* Zapał., Bull. Acad. Cracovie 1913, B. 431; Just’s Bot. Jahresb. xli. II. 176”).

Duplicate (or multiple) publication, in whole or in part, is problematic in the context of botanical nomenclature. The verification of the dates of Zapałowicz’s publications is critical because of the principle of priority: the earliest place and date of valid publication of a new name is the correct one and later redundant publication(s) of names has(ve) no nomenclatural standing. Therefore, we undertook ancillary bibliographic studies to clarify the correct dates of publication for each of Zapałowicz’s works. As an addition to Taxonomic Literature II (Stafleu and Cowan 1988) records, the dates of publication of the component parts of Zapałowicz’s *Conspectus* are here confirmed or revised. We also verified the data available in IPNI related to Zapałowicz’s names of species and nothospecies (IPNI 2019), including verification of a few names published outside the *Conspectus*.

## Material and methods

We reviewed materials housed in the Jagiellonian Library (the *Rozprawy*, Vols. 4B–14B; the bookselling catalogue of the Spółka Wydawnicza Polska [= Polish Publishing Company] in the collection Documents of Everyday Life, Vols. 1904–1912; the bookselling catalogue of Gebethner and Co. Publishing House, digital copies accessed via Jagiellonian Digital Library, Vols. 1904–1911; accession books of the Jagiellonian Library from 1904–1914 in the collection of the Manuscript Section), in the Scientific Library of the PAAS and Polish Academy of Sciences (PAS) (the *Rozprawy*, Vols. 4B to 14B; the *Bulletin*, Vols. 1904–1914) and in the library of the W. Szafer Institute of Botany, PAS (the *Rozprawy*, Vols. 4B–14B; the *Bulletin*, Vols. 1904–1914), all located in Kraków, Poland. Additionally, we reviewed the following library collections: Ernst Mayr Library of the Museum of Comparative Zoology at Harvard University, Smithsonian Libraries and the LuEsther T. Mertz Library of the New York Botanical Garden (the *Bulletin*, Vols. 1904–1909, 1911–1914; digital copies accessed via the Biodiversity Heritage Library (BHL, biodiversitylibrary.org), September 2019). We consulted additional libraries in several countries for the presence in their depositories of wrappers (i.e. temporary covers usually disposed of by the binders) of the fascicles of the *Rozprawy* from volumes 4B–14B.

We follow journal abbreviations provided in the database BPH Online (Hunt Institute for Botanical Documentation 2019). If a title is given in two languages, the second title is preceded by a dash (–). Titles (generally English translations) in square brackets and preceded by the equals sign [=] were determined by us, not the original author. For the Polish “zeszyt” of the *Rozprawy*, we generally use the English equivalent “fascicle” and, for the French “livraison” of the *Bulletin*, we use “issue”.

## Results and discussion

### Determination of publication dates

A multilingual international journal, the *Bulletin* and a Polish-language journal, the *Rozprawy*, were edited by the AAS and both consisted of papers presented at the meetings of the Class of Mathematics and Natural Sciences of the AAS. We first searched for explicit evidence about the publication dates (imprint date) of these journals within them. In the *Bulletin*, the publication date of each issue, precisely to the day, is specified in the work itself. The *Rozprawy* volumes, apart from the imprint date at the foot of the title page, labelled each paper according to the date of presentation at the AAS meeting (Suppl. material 1: Table S1). These presentation dates cannot be accepted as publication dates according to Article 31.1 of the ICN (Turland et al. 2018; Turland 2019). In such situations, it becomes necessary to seek additional information to determine the dates of effective publication. The actual years of publication of the *Rozprawy* volumes may vary from the imprint years at the foot of the title page, since books published in December (and sometimes even November) commonly bear the imprint of the following year and books published in January or February occasionally bear the imprint of the preceding year.

### External sources

In cases where the original work either does not provide information on the precise date of publication or the date it bears is suspected of being inaccurate, we pursued additional evidence, external to the work itself. The newly-published editions of the AAS were recorded and advertised by its Bibliographic Commission in the bibliographic indexes published in two journals. The monthly reports were listed in the section entitled *Bibliografia* [= Bibliography] in the journal *Sprawozdania z Czynności i Posiedzeń Akademii Umiejętności w Krakowie* [= Reports on the AAS’s Activities and Meetings (Kraków)] (hereafter, the *Spraw. AU*). The annual reports were published in the journal *Rocznik Akademii Umiejętności w Krakowie* [= AAS Annual (Kraków)] (hereafter, the *Rocznik AU*). In the former journal, the publications most frequently were dated to the nearest month; rarely a range of dates was given (e.g. August–October 1905). In the latter journal, the publications were recorded from 1 May to 30 April of the following year. These records are a rich source of bibliographical information about the publications of the AAS, including signature size, publication prices and, in the case of journals, the table of contents with page numbers. In addition, the notification of the new publications at the sessions of the Class of Mathematics and Natural Sciences of the AAS were noted in years 1912–1914 in the section *Sprawozdania z posiedzeń* [= Reports from the meetings].

All Zapałowicz’s works considered here were printed at the Jagiellonian University Printing House (currently Jagiellonian University Press); therefore, we searched the printing house’s 1904–1914 bill books, which are preserved in the Archives of the Jagiellonian University (ref. codes DUJ 185−DUJ 193), to determine the date of the printing of the *Rozprawy* volumes in years 1904–1914. We found that bills were usually passed from the printing house to the AAS three times per year and dozens of bills for several journals of the AAS were written with the same date. We also checked handwritten minutes of meetings of the Class of Mathematics and Natural Sciences of the AAS from the years 1904–1914, which are housed at the Archives of Science of the PAS and the PAAS, to determine the publication dates of the individual fascicles that comprise volumes of the Rozprawy in years 1904–1914.

The publications of the AAS were distributed by the Spółka Wydawnicza Polska [= Polish Publishing Company] and Gebethner and Co. Publishing House (Stachowska 1973; Gruca 1993). Therefore, we searched their bookselling catalogues, reference copies of which are stored in the collection Documents of Everyday Life of the Jagiellonian Library.

Finally, dates of receipt or accession of the published journal by institutions, societies or museums provide absolute evidence of the latest date that a particular work was published. We searched the accession books of the Jagiellonian Library for dates of receipt of the *Rozprawy* in years 1904–1914. We also located such information for the Tromsø Museum library, which published similar reports in the journal *Tromsø Museum Aarsberetning* [= Tromsø Museum Reports].

### Discussion on particular series

1A. *Bulletin International de l’Académie des Sciences de Cracovie. Classe des Sciences Mathématiques et Naturelles* – *Anzeiger der Akademie der Wissenschaften in Krakau. Mathematisch-Naturwissenschftliche Klasse* (Bull. Int. Acad. Sci. Cracovie, Cl. Sci. Math.) (1901–1909); continued, in part, by

1B. *Bulletin International de l’Académie des Sciences de Cracovie. Classe des Sciences Mathématiques et Naturelles. Série B. Sciences Naturelles* – *Anzeiger der Akademie der Wissenschaften in Krakau*: *Mathematisch*-*Naturwissenschftliche Klasse*. *Reihe B. Biologische Wissenschaften* (Bull. Int. Acad. Sci. Cracovie, Cl. Sci. Math., Sér. B, Sci. Nat.) (1910–1918)

This journal was characterised by frequent title changes at the turn of the 19^th^ and 20^th^ centuries. Initially, there were two international journals founded and administered by Polish scientists in which botanical papers were published. These were a French-language journal, *Bulletin International de l’Académie des Sciences de Cracovie* (1890–1901) and a German-language journal, *Anzeiger der Akademie der Wissenschaften in Krakau* (1890–1901). These two journals were merged in 1902 and the new multilingual journal with a bilingual title was published until 1909. At the same time, the journal was divided into classes; botanical papers went into *Classe des Sciences Mathématiques et Naturelles* – *Mathematisch*-*Naturwissenschaftliche Klasse*. Beginning in 1910, the journal was divided into two series; botany was included in *Série B. Sciences Naturelles* – *Reihe B. Biologische Wissenschaften*. These two series consisted of research summaries (some of them up to two signatures long) that were intended for an international audience. The *Bulletin* was an important platform for Polish scientists for quick dissemination of their scientific research results abroad (Stachowska 1973). In the case of Zapałowicz, a subset of his nomenclatural and taxonomic novelties was excerpted from the Rozprawy prior to publication and printed in the *Bulletin*.

The *Bulletin* used the years of the covered AAS meetings as volume numbers and its issues were numbered starting at one in each year. In all cases except Volume 1907, published in 1907, the volume number (year) preceded the year of its completion (e.g. Volume 1905 was issued in 1906). Each volume consisted of papers presented during a calendar year at ten monthly meetings of the Class of Mathematics and Natural Sciences of the AAS. Consequently, its volumes each comprised ten issues, appearing after every meeting and each issue comprised papers presented there. Beginning in 1910, the issues were printed in 16-page signatures (in *octavo*), with total pages per issue being multiples of 16 (16, 32, 48 etc.). Given this, the published papers could be divided between two adjacent signatures that were published separately. In issues of volumes 1904 to 1909, the date is given on the last text page of the issue, whereas in issues of volumes from 1910 to 1914, the date is given on the verso of the front wrapper. In the *Bulletin*, the title-page date of the volume represents the data of completion of the multipart work, whereas the individual parts were published on earlier dates. In the *Bulletin*, date research is easiest with sets in which the issue wrappers were preserved by the binders, either bound in place or sometimes at the end of a volume. However, the wrappers were often discarded when a volume was complete and sent for binding. In many libraries, no wrappers have been preserved, for example, in the Library of the PAAS and PAS and in the Jagiellonian Library. In the library of the W. Szafer Institute of Botany PAS, however, the wrappers are bound at the end of a volume and some unbound issues in the original wrappers are also housed there. Copies available online in the Biodiversity Heritage Library include the complete issue wrappers bound in place.

The 30 excerpts from Zapałowicz’s work, written in French and Latin, were issued in the *Bulletin* in years 1904 (Vol. 1904, Issue 4) to 1914 (Vol. 1914, Issue 4B). Two parts (23 and 27) were further subdivided between two adjacent signatures and published separately (for details see Suppl. material 1: Table S1). The first part was titled *Uwagi krytyczne nad roślinnością Galicyi* – *Remarques critiques sur la flore de la Galicie*, while the following ones, from the second to the thirtieth, were titled *Krytyczny przegląd roślinności Galicyi* – *Revue critique de la flore de la Galicie*, with accompanying part numbers in Roman numerals (see Appendix I).

In the excerpts, Zapałowicz published 72 new names of species and nothospecies and more than 80 names of infraspecific taxa, accompanied almost always by Latin descriptions and/or diagnoses. In one case, Gypsophila paniculata L. subsp. lithuanica Zapał., there is only a short diagnosis in French but, because the publication dates to before 1935, this French diagnosis does not preclude valid publication of that name. For a list of taxa published in the excerpts, see Suppl. material 1: Table S1. Zapałowicz’s work published in the *Bulletin* preceded its publication in serial form in the *Rozprawy* (see below) and, therefore, publication dates of names that first appeared in the *Bulletin* are the relevant ones for nomenclatural purposes.

2. *Rozprawy Wydziału Matematyczno*-*Przyrodniczego Akademii Umiejętności*, *Dział B. Nauki Biologiczne* (*Seria 3*) (Rozpr. Wydz. Mat.-Przyr. Akad. Umiejętn., Dział B, Nauki Biol.) (1901–1919)

This Polish journal ran from 1901 to 1919 in its third series, as volumes 1B–18B or volumes 41B–58B of the journal as a whole. Here, we use the former numbering. Each volume of the journal comprised full-text versions of the selected papers presented during a particular calendar year at the meetings of the Class of Mathematics and Natural Sciences of the AAS. Papers accepted for publication in the journal, were printed in 16-page signatures (in *octavo*) with continuous pagination. Zapałowicz’s work was issued there in 30 parts in Polish with Latin descriptions/diagnoses and taxonomical remarks. The parts were published in volumes 4B to 14B(1) and titled *Conspectus florae Galiciae criticus* – *Krytyczny przegląd roślinności Galicyi* with respective part number (see Appendix I and Suppl. material 2: Table S2).

**Figure 2.**
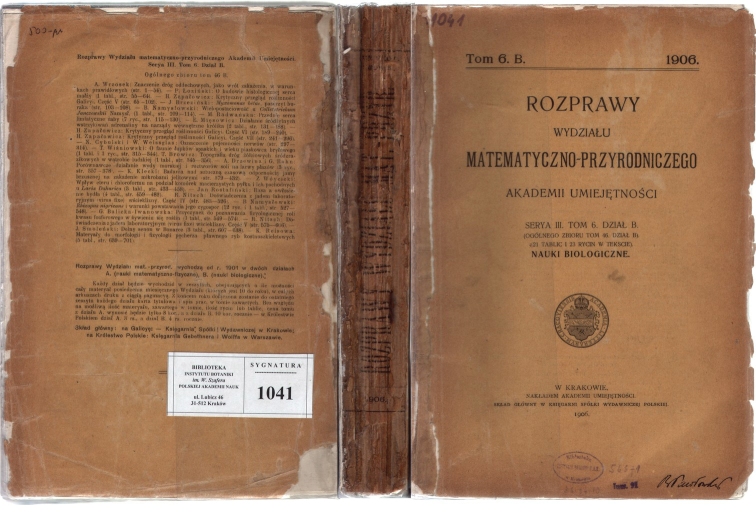
The illustration of binding of Volume 6B of the *Rozprawy Wydziału Matematyczno*-*Przyrodniczego Akademii Umiejętności*, *Dział B. Nauki Biologiczne* (*Seria 3*) showing its book spine and the outside covers, with the 1906 date at the foot of the front cover. Copy from the library of the W. Szafer Institute of Botany, Polish Academy of Sciences (Kraków, Poland).

Information for subscribers provided by the journal itself, on the preserved wrappers of some full year’s volumes, i.e. 1B, 2B, 3B, 4B, 6B (see Fig. 2), 7B and 8B, on their outside back covers, indicated that its volumes would be published in fascicles. The number of these fascicles was never determined, suggesting it varied from one year to another. This information is absent on the wrappers from Volume 12B onwards, which suggests that this practice had been abandoned. Moreover, on these wrappers, the contents of the preceding volumes were listed, followed by the content of the current volume (see Fig. 2), where sometimes division into fascicles was indicated. In Volume 8B, no information on its division into fascicles is presented on its outside back cover. The information about division into fascicles might also have been determined from the individual covers (fascicle wrappers) of the relevant separate fascicles, but few of them have survived.

Although the *Rozprawy* was, at least in several years, published and sent to subscribers in fascicles, the basic sale unit was the journal volume, one per year. After a volume had been completed, it was no longer available in fascicles. The paperback or hardcover copies from the period 1904–1914, to which we had access, do not contain any information that allows recognition of their division into fascicles and their arrangement (except in part volumes 7B and 8B – see Suppl. material 3: Table S3). As there was no fixed schedule for fascicle publication, their frequency of publication is unknown. Our library research showed that some libraries probably received complete volumes of this journal, rather than individual fascicles, because we found only the original paperback volume wrappers in those collections. For example, the library of the W. Szafer Institute of Botany PAS has several volumes of the *Rozprawy* in the original wrappers (Fig. 2) and the British Library houses volumes of the *Rozprawy* with wrappers bound at the end of volumes. Thus, the fascicle compositions and their exact dates of publication are difficult to establish with confidence. The only date is the year (imprint year) provided at the foot of the title page of the complete year volume.

An annotated bibliography of literature relating to botany published by the AAS and PAAS was compiled by Köhler (2004). However, the actual publication dates of volumes of the *Rozprawy* given by him are inaccurate (for details see Suppl. material 2: Table S2). Thus far, there have been a few studies dealing with publication dates of the parts of Zapałowicz’s *Conspectus* issued in the *Rozprawy*. Recently, Wacławska-Ćwiertnia and Mitka (2016) and Paszko et al. (2020) attempted to establish the date of publication for Parts 12 and 13 of the *Conspectus* that appeared in Volume 8B of the *Rozprawy* with imprint year 1909. Based on the bibliographic information from the *Spraw. AU*, Volume 8B was issued in March 1909 (Suppl. material 2: Table S2). Therefore, in the case of this volume, the year provided at its title page is reliable. Wacławska-Ćwiertnia and Mitka (2016) located a copy of Volume 8B at the Jagiellonian Library (ref. no. 284061/III, not 28061/III as given in their paper). This copy of Volume 8B is the only one of these published in 1904–1914 that preserves the original front wrappers (partly damaged) of Fascicles 1 and 3 (see fig. 4 in Paszko et al. 2020). However, no precise (day) dates are provided on these two fascicles; they are dated only 1908 and 1909, respectively. According to the contents listed on its wrapper, Fascicle 1 included eight papers, amongst them Parts 12 and 13 of the *Conspectus* (Zapałowicz 1909a, b). Volume 2 of the book version of the *Conspectus* (Zapałowicz 1908; see below), which comprises the text of these two parts, also bears the imprint year 1908. As none of this information clarifies which source was published first, Wacławska-Ćwiertnia and Mitka (2016) searched the 1908 bill book of the Jagiellonian University Printing House (ref. code DUJ 188). They concluded, based on review of three printers’ receipts from 1908, that three fascicles of Volume 8B of the *Rozprawy* were published in 1908. Fascicle 1 was dated by Wacławska-Ćwiertnia and Mitka (2016) as 5 May 1908. Paszko et al. (2020) considered this inference improbable, because Part 13 of the *Conspectus* was presented at the meeting of the AAS only one day earlier, on 4 May 1908. In addition, Paszko et al. (2020) found, based on the table of contents presented on the verso of the front wrapper of Fascicle 1 and the known number of pages of its last article, that Fascicle 1 comprised 256 pages in sixteen 16-page signatures. These 16 signatures were billed by the printers on two different dates in 1908, 5 May (signatures nos. 1–10 on receipt no. 2404) and 7 August (signatures nos. 11–16 on receipt no. 2577) (Suppl. material 3: Table S3). Thus, Paszko et al. (2020) concluded that the first fascicle could not have been published on the date suggested by Wacławska-Ćwiertnia and Mitka (2016).

The exact date of these fascicles notwithstanding, Paszko et al. (2020) confirmed the order in which the two publications in question had been distributed. They traced information confirming that Fascicle 1 of Volume 8B of the *Rozprawy*, as defined above, was available for sale as early as July–August 1908 in the bookshop of Gebethner and Co. Publishing House, based on its publisher’s catalogue (Świszczowski 1908a), which also provides the table of contents of this fascicle. Recommendation 31A of the ICN helps here, by advising that the date on which the publisher or publisher’s agent delivers printed matter to one of the usual carriers for distribution to the public should be accepted as its date of effective publication (Turland et al. 2018; Turland 2019). Thus, the date of effective publication for Fascicle 1 of Volume 8B of the *Rozprawy* is July–August 1908. Based on the bibliographic information from the *Spraw. AU*, Volume 2 of the *Conspectus* was published in August–October 1908 and was available for sale at Gebethner and Co. in September–October 1908 (Świszczowski 1908b). Therefore, Paszko et al. (2020) confirmed that Parts 12 and 13 of the *Conspectus* were effectively published in the *Rozprawy*.

We have made efforts to determine the publication dates of other volumes of the *Rozprawy*, i.e. Vols. 4B−14B(1). Searches were conducted in several secondary sources (see Suppl. material 2: Table S2), that help determine true dates of publications of these volumes. The *Spraw. AU* is the best source of evidence for the publication dates of the complete volumes of the *Rozprawy* for nomenclatural purposes. We have uncovered considerable evidence that the issuing of volumes of the *Rozprawy* was often delayed. All complete volumes were published at the beginning of the following year (often in March, rarely later), except Volume 14B, which was published in two parts. We found that, in six cases, the imprint years for complete volumes of the *Rozprawy* (and thus the effective publication dates for several parts of the *Conspectus* published there) are not the true dates of publication in this journal; Volumes 4B, 6B, 7B, 11B, 12B and 13B bore the imprint of the preceding year. In most volumes of *Rozprawy* (4B, 5B, 6B−8B (in part) and 10B−14B), the date ranges from the *Spraw. AU* are indicated by us as the publication dates of these journal volumes (see Suppl. material 2: Table S2). The publication of some the volumes of the *Rozprawy* was announced in the Meeting Report section in the *Spraw. AU* (Vols. 11B−14B), but these dates fall within the period known from the Bibliographic section (see Appendix I and Suppl. material 2: Table S2).

We have been unable to find dated fascicle wrappers of the *Rozprawy*, except for Fascicles 1 and 3 of Volume 8B, mentioned above. We still have little knowledge of how the published fascicles of the *Rozprawy* were disseminated to subscribers. We found, as mentioned above, that some fascicles of the volumes of the *Rozprawy* (Vols. 6B−8B) were available for sale in the bookshop of Gebethner and Co. Publishing House, based on its publisher’s catalogue (see Appendix I and Suppl. material 2: Table S2). We determined that Parts 5, 6, 7 (p.p.), 8−10, 12 and 13 of the *Conspectus* from the *Rozprawy* were available for readers in fascicles, prior to the whole appearing in print as a journal volume. Thus, the dates of effective publication for these parts are based on Gebethner and Co. catalogue (for details see Suppl. material 2: Table S2).

In a search for precise information regarding the dates of publication of the subsequent parts of the *Conspectus* published in the *Rozprawy*, we looked through bill books for years 1904–1914 of the Jagiellonian University Printing House (ref. codes DUJ 185−DUJ 193) (see Suppl. material 3: Table S3). We found that signatures were printed throughout the whole calendar year; however, the final ones were printed and postpress operations were carried out at the beginning of the following year (see Suppl. material 3: Table S3 for details).

We found that up to two months from the date of publication given in the *Spraw. AU*, the complete volumes of the *Rozprawy* were entered into the inventory of the Jagiellonian Library: Volume 5B on 19 November 1906, Volume 6B on 23 May 1907 and Volume 7B on 7 May 1908. For the Tromsø Museum, we obtained this type of information from the reports in the journal *Tromsø Museum Aarsberetning* available online, but these dates are later than those for the Jagiellonian Library. In both places, the complete volumes are indexed; however, the precise date (to the day) is limited to a short period of time; in the case of Jagiellonian Library, it covers only the years presented above. The receipt dates are the latest dates of possible publication, but they confirm the earlier dates given in the *Spraw. AU* (see Suppl. material 2: Table S2).

3. *Conspectus florae Galiciae criticus* – *Krytyczny przegląd roślinności Galicyi* (3 Vols) (1906–1911), publication in book form

Three volumes of the *Conspectus* were completed and issued in 1906, 1908 and 1911 (Zapałowicz 1906b, 1908, 1911b). The publication process of the fourth volume was interrupted by the outbreak of the World War I and it was never published (Köhler 2015). The first 21 parts of the *Conspectus* from the serial form in the *Rozprawy* were brought together and published in three volumes. They were reprinted (or preprinted in some cases) with pagination that differed from that used in the *Rozprawy*. The volumes are supplemented by Addenda, Corrigenda and Index generum sections at the end of each volume. The Addenda sections include omitted species with numbers, additional infraspecific forms, new names, a few new species descriptions and additional distribution data. Volume 1 covers Parts 1−7 and the Addenda and Corrigenda to Volume 1, Volume 2 covers Parts 8−13 and the Addenda and Corrigenda to Volumes 1 and 2 and Volume 3 covers Parts 14−21 and the Addenda and Corrigenda to Volumes 1, 2 and 3.

We determined, based on bibliographic data available in the *Spraw. AU*, that the volumes of the *Conspectus* were already available for readers during the following periods: August–October 1906 (Volume 1), August–October 1908 (Volume 2) and November 1911 (Volume 3). The dates given in the publisher’s catalogue of Gebethner and Co. Publishing House are the same or later (Świszczowski 1906, 1908b, 1912). From this, we conclude that the imprint dates at the foot of the title pages were provided correctly in these three volumes. The *Spraw. AU* revealed precise dates of publication for Zapałowicz’s books of the *Conspectus* (Suppl. material 4: Table S4).

Volume 4, which covers parts 22−30 along with further addenda, was not effectively published nor distributed widely. Two printed copies, both lacking covers and title pages, were traced by Köhler (2015), one at the Library of the Jagiellonian University (ref. no. 80990 II) and one at the Library of the Nicolaus Copernicus University at Toruń (ref. no. DT 003208), Poland. Köhler (2015) identified these books as Zapałowicz’s 4^th^ volume based on the signature mark “H. Zapałowicz T. IV.” (“T” standing for Polish “Tom” = volume) printed on the bottom of the first page of each signature (see fig. 9 in Köhler 2015). A digitised version of Toruń copy has been available online since 11 March 2015 in Djvu format from the collections of Kujawsko-Pomorska Biblioteka Cyfrowa [= Kujawsko-Pomorska Digital Library] (http://www.kpbc.ukw.edu.pl/dlibra). This electronic material does not constitute effective publication, because it was not published in Portable Document Format (PDF) and does not have an International Standard Serial Number (ISSN) or an International Standard Book Number (ISBN) (see Article 29.1 of the ICN, Knapp et al. 2011; Turland et al. 2018). From a practical point of view, this affects only those names that were introduced (or their descriptions amended) in the Addenda sections of this volume, as they were published nowhere else.

In the prefaces to Zapałowicz’s books (Zapałowicz 1906b, 1908, 1911b), there are notes that each volume represents the collective reprint from the relevant volumes of the *Rozprawy*. This is almost always true with regard to their contents; however, we detected a small paragraph (11 text lines) related to Dianthus armeria L. var. dubius Zapał. (Zapałowicz 1911b: 109) that appeared in Volume 3, but is missing at the respective place in the *Rozprawy* (Zapałowicz 1911a: 682). This may suggest that the respective signatures in the *Rozprawy* and the book were printed simultaneously. Alternatively, the book signatures may have been printed sometimes even before those for the *Rozprawy*, which may explain why the text noted above is missing in the *Rozprawy*, even though the journal was supposed to be the original publication.

We paid special attention to “reprints” from the journal with possible earlier appearance dates than those of the *Rozprawy*. Our research revealed that more than half of the material in book form (Zapałowicz 1906b, 1908, 1911b) appeared earlier in serial form in the *Rozprawy*. This situation concerns 13 parts (i.e. Parts 1–3, 8–17) of the book, which we considered to be reprints. Seven parts appeared earlier in book form (i.e. Parts 5–7, 18–21) and these parts are considered by us to be original. The publication dates of Part 4 (Zapałowicz 1906a) are the same for both sources; we therefore recommend that both be cited for names published in Part 4 (for details, see Suppl. material 4: Table S4).

As mentioned above, some of Zapałowicz’s names, supplementing those from serial form in the *Rozprawy*, were published exclusively at the ends of book volumes of the *Conspectus* in addenda to the current and the previously-issued volumes. These names must be cited from the relevant book volume of the *Conspectus* (Zapałowicz 1906b, 1908, 1911b).

### *Conspectus florae Galiciae criticus* and other sources online

Zapałowicz’s works are available for readers via the Polona website (polona.pl). The Biodiversity Heritage Library (BHL) (www.biodiversitylibrary.org) has digitised several of the volumes of the *Bulletin*, together with the covers accompanying each issue. Other sources are available as follows: the *Rozprawy* in the Wielkopolska Digital Library (www.wbc.poznan.pl), the *Spraw. AU* in the Silesian Digital Library (www.sbc.org.pl), the journal *Rocznik AU* on the RCIN platform (rcin.org.pl) and Gebethner and Co. publisher’s catalogue titled *Katalog Nowych Książek* [= Catalogue of New Books] in the Jagiellonian Digital Library (jbc.bj.uj.edu.pl/dlibra).

### Updates to the International Plant Names Index (IPNI) database

The IPNI database provides nomenclatural information (spelling, author, types and first place and date of publication) for the scientific names of vascular plants. However, not all information concerning Zapałowicz’s names is accurate in its current version. Entries of Zapałowicz’s names at species rank (including hybrids) in IPNI are corrected here and the hope is expressed that the current inventory may be useful for fixing them.

A search of names in IPNI brought to light more than one hundred plant names for species and hybrids described by Zapałowicz (IPNI 2019) (for details, see Table 1). Amongst these records, we have identified 14 duplicate IPNI entries that are records of the same names with two different bibliographic citations. They probably derived from more than one of the three original source databases (Index Kewensis, the Gray Card Index and the Australian Plant Names Index) that had been combined in the late 1990s to create IPNI (Croft et al. 1999). It seems that the deduplication process conducted by the IPNI team in early 2016 (Nic Lughadha et al. 2016) was not fully successful, especially in cases when we deal with multiple data sources for Zapałowicz’s names. After our processing of the mentioned IPNI entries, we obtained 98 names at species rank (including hybrids). We have searched for their first place and date of publication. Two names, Rorippa × wimmeri Zapał. and *Viola berdaui* Zapał., omitted in IPNI, are added by us. One name, Viola × roxolanica attributed to Zapałowicz, must be deleted. This taxon was described by Błocki (Deutsche Bot. Monatsschr. 5: 147. 1887) at species rank, then Zapałowicz transferred it to a hybrid category. Therefore, it must be cited as Viola × roxolanica Błocki (pro sp.). In total, we obtained 99 names at species rank (including hybrids), attributed to Zapałowicz, including one combination (*Rumex carpaticus* (Zapał.) Zapał.). Most of these names, 94 out of 99, are attributed to the *Conspectus*. Corrections are here provided for more than 60% of the respective IPNI entries. We have checked their name spelling (four corrections), authorships (three corrections), associated bibliographical details (63 corrections) and journal names (or their abbreviations; for details, see Table 1).

**Table 1.** The inventory of bibliographic data covering Zapałowicz’s names of species and hybrids in the International Plant Names Index (IPNI) database. Species and nothospecies names, authorship and place of their publications were corrected, where necessary, according to the rules of the Shenzen Code (Turland & al. in Regnum Veg. 159. 2018). Major source corrections are indicated by the word “yes”. The list of taxa names with associated basic bibliographical details was extracted on 10 July 2019 from the IPNI database. Duplicated names are marked for deletion.

No.	Taxon name	Authorship	Bibliographic information from the current IPNI database	Source correction needed	Revised bibliographic information by the present authors	Remarks
**The IPNI entries attributed to Hugo Zapałowicz**						
**1**	***Aconitum × berdaui***	Zapał.	Aconitum × berdaui Zapał., Consp. Fl. Galic. Crit. 2: 229 (1908).	yes	Bull. Int. Acad. Sci. Cracovie, Cl. Sci. Math. 1908(3): 143. 1908	
**2**	***Aconitum × bucovinense***	Zapał.	*Aconitum bucovinense* Zapał., Consp. Fl. Galic. Crit. ii. 230 (1908).	yes	Bull. Int. Acad. Sci. Cracovie, Cl. Sci. Math. 1908(3): 144. 1908	
**3**	***Alsine zarencznyi***	Zapał.	*Alsine zarencznyi* Zapał., Consp. Fl. Galic. Crit. iii. 25 (1911).	yes		Duplicate entry to be deleted.
			*Alsine zarenczyni* Zapał., Bull. Int. Acad. Sci. Cracovie, Cl. Sci.Math., Ser. B, Sci. Nat. 1910, 168.		Bull. Int. Acad. Sci. Cracovie, Cl. Sci. Math., Ser. B, Sci. Nat. 1910(3B): 168. 1910	Spelling in IPNI incorrect: “*zarenczyni*” should be changed to “*zarencznyi*”
**4**	***Alyssum borysthenicum***	Zapał.	*Alyssum borysthenicum* Zapał., Bull. Acad. Cracovie 1912, B, 710.		Bull. Int. Acad. Sci. Cracovie, Cl. Sci. Math., Ser. B, Sci. Nat. 1912(7B): 710. 1912	
**5**	***Alyssum brodense***	Zapał.	*Alyssum brodense* Zapał., Bull. Acad. Cracovie 1912, B, 711.		Bull. Int. Acad. Sci. Cracovie, Cl. Sci. Math., Ser. B, Sci. Nat. 1912(7B): 711. 1912	
**6**	***Arabis besseri***	Zapał.	*Arabis besseri* Zapał., Bull. Acad. Cracovie 1912, B. 17.		Bull. Int. Acad. Sci. Cracovie, Cl. Sci. Math., Ser. B, Sci. Nat. 1912(2B): 17. 1912	
**7**	***Arabis × calcigena***	Zapał.	*Arabis calcigena* Zapał., Bull. Acad. Cracovie 1912, B. 21, hybr.		Bull. Int. Acad. Sci. Cracovie, Cl. Sci. Math., Ser. B, Sci. Nat. 1912(2B): 21. 1912	
**8**	***Arabis × decipiens***	Zapał.	*Arabis decipiens* Zapał., Bull. Acad. Cracovie 1912, B. 20, hybr.		Bull. Int. Acad. Sci. Cracovie, Cl. Sci. Math., Ser. B, Sci. Nat. 1912(2B): 20. 1912	
**9**	***Arabis × kotulae***	Zapał.	*Arabis kotulae* Zapał., Bull. Acad. Cracovie 1912, 11. 21, hybr.		Bull. Int. Acad. Sci. Cracovie, Cl. Sci. Math., Ser. B, Sci. Nat. 1912(2B): 21. 1912	
**10**	***Arabis × saccata***	Zapał.	*Arabis saccata* Zapał., Bull. Acad. Cracovie 1912, B. 22, hybr.		Bull. Int. Acad. Sci. Cracovie, Cl. Sci. Math., Ser. B, Sci. Nat. 1912(2B): 22. 1912	
**11**	***Atriplex polonicum***	Zapał.	*Atriplex polonicum* Zapał., Consp. Fl. Galic. Crit. ii. 169 (1908).	yes	Bull. Int. Acad. Sci. Cracovie, Cl. Sci. Math. 1907(10): 1080. 1907	
**12**	***Bromus janczewskii***	Zapał.	*Bromus janczewskii* Zapał., Bull. Acad. Cracovie 1904, 306.		Bull. Int. Acad. Sci. Cracovie, Cl. Sci. Math. 1904(6): 306. 1904	
			*Bromus janczewskii* Zapał., Consp. Fl. Galic. Crit. i. 73 (1906).	yes		Duplicate entry to be deleted.
**13**	***Bunias dubia***	Zapał.	*Bunias dubia* Zapał., Bull. Acad. Cracovie 1913, B. 446; Just’s Bot. Jahresb. xli. II. 169.		Bull. Int. Acad. Sci. Cracovie, Cl. Sci. Math., Ser. B, Sci. Nat. 1913(7B): 446. 1913	
**14**	***Calamagrostis kotulae***	Zapał.	*Calamagrostis kotulae* Zapał., Bull. Acad. Cracovie 1904, 163.		Bull. Int. Acad. Sci. Cracovie, Cl. Sci. Math. 1904(3): 163. 1904	
			*Calamagrostis kotulae* Zapał., Consp. Fl. Galic. Crit. i. 23 (1906).	yes		Duplicate entry to be deleted.
**15**	***Cardamine × dubia***	Zapał.	*Cardamine dubia* Zapał., Bull. Acad. Cracovie 1912, B, 13, hybr.		Bull. Int. Acad. Sci. Cracovie, Cl. Sci. Math., Ser. B, Sci. Nat. 1912(1B): 13. 1912	
**16**	***Cardamine × tatrensis***	Zapał.	*Cardamine tatrensis* Zapał., Bull. Acad. Cracovie 1912, B, 12, hybr.		Bull. Int. Acad. Sci. Cracovie, Cl. Sci. Math., Ser. B, Sci. Nat. 1912(1B): 12. 1912	
**17**	***Carex × bogdanensis***	Zapał.	*Carex bogdanensis* Zapał., Consp. Fl. Galic. Crit. iii. 233 (1911), hybr.		Consp. fl. Galic. crit. 3. 233. 1911	
**18**	***Carex × paczoskii***	Zapał.	*Carex paczoskii* Zapał., Consp. Fl. Galic. Crit. iii. 234 (1911), hybr.		Consp. fl. Galic. crit. 3. 234. 1911	
**19**	***Carex × raciborskii***	Zapał.	*Carex raciborskii* Zapał., Consp. Fl. Galic. Crit. iii. 233 (1911), hybr.		Consp. fl. Galic. crit. 3. 233. 1911	
**20**	***Cerastium ciarcanense***	Zapał.	*Cerastium ciarcanense* Zapał., Bull. Acad. Sc. Cracovie, Ser. B. 1910, 436.		Bull. Int. Acad. Sci. Cracovie, Cl. Sci. Math., Ser. B, Sci. Nat. 1910(6B): 436. 1910	
			*Cerastium ciarcanense* Zapał., Consp. Fl. Galic. Crit. iii. 90 (1911).	yes		Duplicate entry to be deleted.
**21**	***Cerastium pietrosuanum***	Zapał.	*Cerastium pietrosuanum* Zapał., Bull. Acad. Sc. Cracovie, Ser. B. 1910, 436.		Bull. Int. Acad. Sci. Cracovie, Cl. Sci. Math., Ser. B, Sci. Nat. 1910(6B): 436. 1910	
			*Cerastium pietrosuanum* Zapał., Consp. Fl. Galic. Crit. iii. 95 (1911).	yes		Duplicate entry to be deleted.
**22**	***Cerastium raciborskii***	Zapał.	*Cerastium raciborskii* Zapał., Bull. Acad. Sc. Cracovie, Ser. B. 1910, 433.		Bull. Int. Acad. Sci. Cracovie, Cl. Sci. Math., Ser. B, Sci. Nat. 1910(6B): 434. 1910	
			*Cerastium raciborskii* Zapał., Consp. Fl. Galic. Crit. iii. 84 (1911).	yes		Duplicate entry to be deleted.
**23**	***Cerastium × tatrense***	Zapał.	*Cerastium tatrense* Zapał., Bull. Acad. Sc. Cracovie, Ser. B. 1910, 437.		Bull. Int. Acad. Sci. Cracovie, Cl. Sci. Math., Ser. B, Sci. Nat. 1910(6B): 437. 1910	
			*Cerastium tatrense* Zapał., Consp. Fl. Galic. Crit. iii. 97 (1911), hybr.	yes		Duplicate entry to be deleted.
**24**	***Crocus babiogorensis***	Zapał.	*Crocus babiogorensis* Zapał., Consp. Fl. Galic. Crit. i. 185 (1906).	yes	Bull. Int. Acad. Sci. Cracovie, Cl. Sci. Math. 1906(5): 326. 1906	
**25**	***Delphinium nacladense***	Zapał.	*Delphinium nacladense* Zapał., Consp. Fl. Galic. Crit. ii. 202 (1908).	yes	Bull. Int. Acad. Sci. Cracovie, Cl. Sci. Math. 1908(3): 142. 1908	
**26**	***Dianthus euponticus***	Zapał.	*Dianthus euponticus* Zapał., Consp. Fl. Gallic. Crit. iii. 141 (1911); et in Bull. Acad Crac. 1911, B. 10.	yes	Bull. Int. Acad. Sci. Cracovie, Cl. Sci. Math., Ser. B, Sci. Nat. 1911(1B): 10. 1911	Spelling of the abbreviation in IPNI incorrect: “Gallic.” changed to “Galic.”
**27**	***Dianthus × lacinulatus***	Zapał.	*Dianthus lacinulatus* Zapał., Consp. Fl. Galic. Crit. iii. 161 (1911); et in Bull. Acad. Crac. 1911, B. 163, hybr.	yes	Bull. Int. Acad. Sci. Cracovie, Cl. Sci. Math., Ser. B, Sci. Nat. 1911(3B): 163. 1911	
**28**	***Dianthus polonicus***	Zapał.	*Dianthus polonicus* Zapał., Consp. Fl. Galic. Crit. iii. 122 (1911); et in Bull. Acad. Crac. 1911, B. 7.	yes	Bull. Int. Acad. Sci. Cracovie, Cl. Sci. Math., Ser. B, Sci. Nat. 1911(1B): 7. 1911	
**29**	***Dianthus × zarencznianus***	Zapał.	*Dianthus zarencznianus* Zapał., Consp. Fl. Galic. Crit. iii. 149 (1911); et in Bull. Acad. Crac. 1911, B. 162, hybr.	yes	Bull. Int. Acad. Sci. Cracovie, Cl. Sci. Math., Ser. B, Sci. Nat. 1911(3B): 162. 1911	
**30**	***Diplotaxis polonica***	Zapał.	*Diplotaxis polonica* Zapał., Bull. Acad. Cracovie 1913, 11. 273; Just’s Bot. Jahresb. 1913, xli. II. 171.	yes	Bull. Int. Acad. Sci. Cracovie, Cl. Sci. Math., Ser. B, Sci. Nat. 1913(5B): 273. 1913	
**31**	***Erysimum hungaricum***	Zapał.	*Erysimum hungaricum* Zapał., Bull. Acad. Cracovie 1913, II. 49; Just’s Bot. Jahresb. 1913, xli. II. 172.	yes	Bull. Int. Acad. Sci. Cracovie, Cl. Sci. Math., Ser. B, Sci. Nat. 1913(3B): 49. 1913	
**32**	***Euphrasia carpatica***	Zapał.	*Euphrasia carpatica* Zapał., Spraw. Komis. Fizjogr. xxiv. 270 (1889).		Spraw. Komis. Fizjogr. 24: 270. 1889; Roślinna szata Gór Pokucko-Marmaroskich 270. 1889	Roślinna szata... is separate from Spraw. Komis. Fizjogr. 24
			*Euphrasia carpatica* Zapał., Spraw. Komis. Fizjogr. xlii. II. 6 (1908).	yes		Duplicate entry to be deleted.
**33**	***Festuca × czarnohorensis***	Zapał.	*Festuca czarnohorensis* Zapał., Consp. Fl. Gallic. Crit. 3: 230 (1911).		Consp. fl. Galic. crit. 3. 230. 1911	Spelling of the abbreviation in IPNI incorrect: “Gallic.” changed to “Galic.”
**34**	***Festuca hackeliana***	Zapał.	*Festuca hackeliana* Zapał., Consp. Fl. Galic. Crit. iii. 231 (1911).		Consp. fl. Galic. crit. 3. 231. 1911	
**35**	***Festuca makutrensis***	Zapał.	*Festuca makutrensis* Zapał., Consp. Fl. Galic. Crit. iii. 229 (1911).	yes		Duplicate entry to be deleted.
						*Festuca makutrensis* in Consp. fl. Galic. crit. 3. 229. 1911 is also recorded with the phrase “m. (n. sp.)”, that is confusing for this name.
			*Festuca makutrensis* Zapał., Kosmos xxxv. 782–786 (1910); cf. Bot. Centralbl. cxvi. 420.		Kosmos (Lvov) 35: 783. 1910	
**36**	***Festuca pietrosii***	Zapał.	*Festuca pietrosii* Zapał., Bull. Acad. Cracovie 1904, 304.		Bull. Int. Acad. Sci. Cracovie, Cl. Sci. Math. 1904(6): 304. 1904	
			*Festuca pietrosii* Zapał., Consp. Fl. Galic. Crit. i. 63 (1906); ii. 306 (1908).	yes		Duplicate entry to be deleted.
**37**	***Festuca × pocutica***	Zapał.	*Festuca pocutica* Zapał., Consp. Fl. Galic. Crit. iii. 230 (1911), hybr.		Consp. fl. Galic. crit. 3. 230. 1911	
**38**	***Festuca polesica***	Zapał.	*Festuca polesica* Zapał., Bull. Acad. Cracovie 1904, 303.		Bull. Int. Acad. Sci. Cracovie, Cl. Sci. Math. 1904(6): 303. 1904	
			*Festuca polesica* Zapał., Consp. Fl. Galic. Crit. i. 62 (1906).	yes		Duplicate entry to be deleted.
**39**	***Festuca polonica***	Zapał.	*Festuca polonica* Zapał., Bull. Acad. Cracovie 1904, 302.		Bull. Int. Acad. Sci. Cracovie, Cl. Sci. Math. 1904(6): 302. 1904	
			*Festuca polonica* Zapał., Consp. Fl. Galic. Crit. i. 60 (1906).	yes		Duplicate entry to be deleted.
**40**	***Heliosperma arcanum***	Zapał.	*Heliosperma arcanum* Zapał., Consp. Fl. Galic. Crit. iii. 203 (1911).	yes	Bull. Int. Acad. Sci. Cracovie, Cl. Sci. Math., Ser. B, Sci. Nat. 1911(6B): 498. 1911	
**41**	***Hesperis carpatica***	Zapał.	*Hesperis carpatica* Zapał., Spraw. Komis. Fizjogr. xxiv. (1889) 106.	yes	Spraw. Komis. Fizjogr. 24: 106. 1889; Roślinna szata Gór Pokucko-Marmaroskich 106. 1889	Roślinna szata... is separate from Spraw. Komis. Fizjogr. 24
**42**	***Hesperis pontica***	Zapał.	*Hesperis pontica* Zapał., Bull. Acad. Cracovie 1912, B, 1158.		Bull. Int. Acad. Sci. Cracovie, Cl. Sci. Math., Ser. B, Sci. Nat. 1912(9B): 1183. 1912	
**43**	***Hieracium zapalowiczii***	Uechtr. ex Zapał.	*Hieracium zapalowiczii* Uchtr. ex Zapał., Kosmos xxxv. 782–786 (1910); cf. Bot. Centralbl. cxvi. 420.	yes	Spraw. Komis. Fizjogr. 39: 37. 1906	Description from Uechtritz’s letter quoted (without the species name) in Spraw. Komis. Fizjogr. 24: 234–235. 1889 (and thus in the separate Roślinna szata Gór Pokucko-Marmaroskich 234–235. 1889). The name, ascribed by Zapałowicz to Uechtritz, appeared for the first time in Spraw. Komis. Fizjogr. 39: 37. 1906 with indication of description in the former source.
**44**	***Iris pontica***	Zapał.	*Iris pontica* Zapał., Consp. Fl. Galic. Crit. i. 191 (1906).	yes	Bull. Int. Acad. Sci. Cracovie, Cl. Sci. Math. 1906(5): 326. 1906	
**45**	***Isatis ciesielskii***	Zapał.	*Isatis ciesielskii* Zapał., Bull. Acad. Cracovie 1913, B. 447; Just’s Bot. Jahresb. 1913, xli. II. 173.	yes	Bull. Int. Acad. Sci. Cracovie, Cl. Sci. Math., Ser. B, Sci. Nat. 1913(7B): 447. 1913	
**46**	***Isatis kamienskii***	Zapał.	*Isatis kamienskii* Zapał., Bull. Acad. Cracovie 1913, B. 447.		Bull. Int. Acad. Sci. Cracovie, Cl. Sci. Math., Ser. B, Sci. Nat. 1913(7B): 447. 1913	
**47**	***Muscari pocuticum***	Zapał.	*Muscari pocuticum* Zapał., Consp. Fl. Galic. Crit. i. 164 (1906).	yes	Bull. Int. Acad. Sci. Cracovie, Cl. Sci. Math. 1906(2): 100. 1906	
**48**	***Papaver corona-sancti-stephani***	Zapał.	*Papaver corona-sti-stephani* Zapał., Bull. Int. Acad. Sci. Cracovie, Cl. Sci. Math., Ser. B, Sci. Nat. 620 (1911).		Bull. Int. Acad. Sci. Cracovie, Cl. Sci. Math., Ser. B, Sci. Nat. 1911(8B): 620. 1911	Original abbreviation “sti” expanded into “sancti” in accordance with Art. 60.14 of ICN
**49**	***Poa janczewskii***	Zapał.	*Poa janczewskii* Zapał., Consp. Fl. Galic. Crit. i. 292 (1906).	yes	Spraw. Komis. Fizjogr. 39: 34. 1906	Zapałowicz in Consp. fl. Galic. crit. 1. 292. 1906 provided proper citation with page number to “Sprawozd. Kom. fiz. 1905 str. [page] 34”.
**50**	***Poa rodnensis***	Zapał.	*Poa rodnensis* Zapał., Consp. Fl. Galic. Crit. ii. 302 (1908).	yes	Spraw. Komis. Fizjogr. 42: 62. 1908	Zapałowicz in Consp. fl. Galic. crit. 2. 302. 1908 provided imprecise citation to Sprawozd. Kom. fiz. vol. XLII, II).
**51**	***Polygonum × asperulum***	Zapał.	*Polygonum asperulum* Zapał., Consp. Fl. Galic. Crit. ii. 145 (1908).	yes	Bull. Int. Acad. Sci. Cracovie, Cl. Sci. Math. 1907(6): 631. 1907	
**52**	***Polygonum × janoviense***	Zapał.	*Polygonum janoviense* Zapał., Consp. Fl. Galic. Crit. ii. 131 (1908).	yes	Bull. Int. Acad. Sci. Cracovie, Cl. Sci. Math. 1907(6): 631. 1907	
**53**	***Pulsatilla × janczewskii***	Zapał.	*Pulsatilla janczewskii* Zapał., Consp. Fl. Galic. Crit. ii. 244 (1908).	yes	Bull. Int. Acad. Sci. Cracovie, Cl. Sci. Math. 1908(5): 448. 1908	
**54**	***Pulsatilla × tarnoviensis***	Zapał.	*Pulsatilla tarnoviensis* Zapał., Consp. Fl. Galic. Crit. ii. 245 (1908).	yes	Bull. Int. Acad. Sci. Cracovie, Cl. Sci. Math. 1908(5): 449. 1908	
**55**	***Ranunculus × gilibertii***	Zapał.	*Ranunculus gilibertii* Zapał., Consp. Fl. Galic. Crit. ii. 289 (1908).	yes	Bull. Int. Acad. Sci. Cracovie, Cl. Sci. Math. 1908(5): 449. 1908	
**56**	***Ranunculus × klukii***	Zapał.	*Ranunculus klukii* Zapał., Consp. Fl. Galic. Crit. ii. 289 (1908).	yes	Bull. Int. Acad. Sci. Cracovie, Cl. Sci. Math. 1908(5): 449. 1908	
**57**	***Rorippa cracoviensis***	Zapał.	*Rorippa cracoviensis* Zapał., Bull. Acad. Cracovie 1912, B, 345.		Bull. Int. Acad. Sci. Cracovie, Cl. Sci. Math., Ser. B, Sci. Nat. 1912(4B): 345. 1912	Original spelling of the genus name “*Roripa*” used by Zapałowicz corrected to “*Rorippa*”.
**58**	***Rorippa × oslawiensis***	Zapał.	*Rorippa oslawiensis* Zapał., Bull. Acad. Cracovie 1912, B, 347, hybr.		Bull. Int. Acad. Sci. Cracovie, Cl. Sci. Math., Ser. B, Sci. Nat. 1912(4B): 347. 1912	Original spelling of the genus name “*Roripa*” used by Zapałowicz corrected to “*Rorippa*”.
**59**	***Rorippa × podolica***	Zapał.	*Rorippa podolica* Zapał., Bull. Acad. Cracovie 1912, B, 346, hybr.		Bull. Int. Acad. Sci. Cracovie, Cl. Sci. Math., Ser. B, Sci. Nat. 1912(4B): 346. 1912	Original spelling of the genus name “*Roripa*” used by Zapałowicz corrected to “*Rorippa*”.
**60**	***Rorippa × sodalis***	Zapał.	*Rorippa sodalis* Zapał.,Bull. Acad. Cracovie 1912, B, 347, hybr.		Bull. Int. Acad. Sci. Cracovie, Cl. Sci. Math., Ser. B, Sci. Nat. 1912(4B): 347. 1912	Original spelling of the genus name “*Roripa*” used by Zapałowicz corrected to “*Rorippa*”.
**61**	***Rorippa × viaria***	Zapał.	*Rorippa viaria* Zapał., Bull. Acad. Cracovie 1912, B, 346, hybr.		Bull. Int. Acad. Sci. Cracovie, Cl. Sci. Math., Ser. B, Sci. Nat. 1912(4B): 346. 1912	Original spelling of the genus name “*Roripa*” used by Zapałowicz corrected to “*Rorippa*”.
**62**	***Rorippa × wimmeri***	Zapał.	[absent]		Rozpr. Wydz. Mat.-Przyr. Akad. Umiejętn., Dział B, Nauki Biol., (Ser. 3) 12B(52B): 179. 1912 [Feb. 1913]	Original spelling of the genus name “*Roripa*” used by Zapałowicz corrected to “*Rorippa*”.
**63**	***Rorippa × wislokiensis***	Zapał.	*Rorippa wislokiensis* Zapał., Bull. Acad. Cracovie 1912, B, 348, hybr.		Bull. Int. Acad. Sci. Cracovie, Cl. Sci. Math., Ser. B, Sci. Nat. 1912(4B): 348. 1912	Original spelling of the genus name “*Roripa*” used by Zapałowicz corrected to “*Rorippa*”.
**64**	***Rumex × babiogorensis***	Zapał.	*Rumex babiogorensis* Zapał., Consp. Fl. Galic. Crit. ii. 116 (1908).	yes	Bull. Int. Acad. Sci. Cracovie, Cl. Sci. Math. 1907(4): 254. 1907	
**65**	***Rumex ×błockii***	Zapał.	*Rumex blockii* Zapał., Consp. Fl. Galic. Crit. ii. 111 (1908).	yes	Rozpr. Wydz. Mat.-Przyr. Akad. Umiejętn., Dział B, Nauki Biol., (Ser. 3) 7B(47B): 292. 1907 [Jan.–Febr. 1908]	
**66**	***Rumex carpaticus***	(Zapał.) Zapał.	*Rumex carpaticus* Zapał., Consp. Fl. Galic. Crit. ii. 118 (1908)		Bull. Int. Acad. Sci. Cracovie, Cl. Sci. Math. 1907(4): 253. 1907	Authority citation in IPNI should be corrected. This name is a combination based on *Rumex arifolius* All. α [var.] *carpaticus* Zapał. (Spraw. Komis. Fizjogr. 24: 285. 1889; Roślinna szata Gór Pokucko-Marmaroskich 285. 1889).
			*Rumex carpaticus* Zapał., Sprawozd. Akad. Umiejętn. Krakow. xlv. III. 153 (1911).	yes		Duplicate entry to be deleted.
**67**	***Salix × cracoviensis***	Zapał.	*Salix cracoviensis* Zapał., Consp. Fl. Galic. Crit. ii. 78 (1908).	yes	Rozpr. Wydz. Mat.-Przyr. Akad. Umiejętn., Dział B, Nauki Biol., (Ser. 3) 7B(47B): 230. 1907 [Jan.–Febr. 1908]	
**68**	***Salix × janczewskii***	Zapał.	*Salix janczewskii* Zapał., Consp. Fl. Galic. Crit. ii. 67 (1908).	yes	Rozpr. Wydz. Mat.-Przyr. Akad. Umiejętn., Dział B, Nauki Biol., (Ser. 3) 7B(47B): 219. 1907 [Jan.–Febr. 1908]	
**69**	***Salix × kotuliana***	Zapał.	*Salix kotuliana* Zapał., Consp. Fl. Galic. Crit. ii, 68 (1908).	yes	Rozpr. Wydz. Mat.-Przyr. Akad. Umiejętn., Dział B, Nauki Biol., (Ser. 3) 7B(47B): 220. 1907 [Jan.–Febr. 1908]	
**70**	***Salix × pocutica***	Zapał.	*Salix pocutica* Zapał., Consp. Fl. Galic. Crit. ii. 33 (1908).	yes	Rozpr. Wydz. Mat.-Przyr. Akad. Umiejętn., Dział B, Nauki Biol., (Ser. 3) 7B(47B): 185. 1907 [Jan.–Febr. 1908]	
**71**	***Salix × polesica***	Zapał.	*Salix polesica* Zapał., Consp. Fl. Galic. Crit. ii. 76 (1908).	yes	Rozpr. Wydz. Mat.-Przyr. Akad. Umiejętn., Dział B, Nauki Biol., (Ser. 3) 7B(47B): 228. 1907 [Jan.–Febr. 1908]	
**72**	***Salix × rehmanii***	Zapał.	*Salix rehmani* Zapał., Consp. Fl. Galic. Crit. ii. 41 (1908).	yes	Rozpr. Wydz. Mat.-Przyr. Akad. Umiejętn., Dział B, Nauki Biol., (Ser. 3) 7B(47B): 193. 1907 [Jan.–Febr. 1908]	In accordance with Art. 60.8 of ICN, the specific epithet “*rehmani*” is corrected to “*rehmanii*”.
**73**	***Salix × sandomiriensis***	Zapał.	*Salix sandomiriensis* Zapał., Consp. Fl. Galic. Crit. ii. 75 (1908).	yes	Rozpr. Wydz. Mat.-Przyr. Akad. Umiejętn., Dział B, Nauki Biol., (Ser. 3) 7B(47B): 228. 1907 [Jan.–Febr. 1908]	
**74**	***Salix × sarmatica***	Zapał.	*Salix sarmatica* Zapał., Consp. Fl. Galic. Crit. ii. 56 (1908).	yes	Rozpr. Wydz. Mat.-Przyr. Akad. Umiejętn., Dział B, Nauki Biol., (Ser. 3) 7B(47B): 208. 1907 [Jan.–Febr. 1908]	
**75**	***Salix tatrorum***	Zapał.	*Salix tatrorum* Zapał., Consp. Fl. Galic. Crit. ii. 65 (1908).	yes	Bull. Int. Acad. Sci. Cracovie, Cl. Sci. Math. 1907(2): 59. 1907	
**76**	***Salix × vistulensis***	Zapał.	*Salix vistulensis* Zapał., Consp. Fl. Galic. Crit. ii. 77 (1908).	yes	Rozpr. Wydz. Mat.-Przyr. Akad. Umiejętn., Dział B, Nauki Biol., (Ser. 3) 7B(47B): 229. 1907 [Jan.–Febr. 1908]	
**77**	***Salix × volhyniensis***	Zapał.	*Salix volhyniensis* Zapał., Consp. Fl. Galic. Crit. ii. 75 (1908).	yes	Rozpr. Wydz. Mat.-Przyr. Akad. Umiejętn., Dział B, Nauki Biol., (Ser. 3) 7B(47B): 227. 1907 [Jan.–Febr. 1908]	
**78**	***Salix ×wołoszczakii***	Zapał.	*Salix woloszczakii* Zapał., Consp. Fl. Galic. Crit. ii. 40 (1908).	yes	Rozpr. Wydz. Mat.-Przyr. Akad. Umiejętn., Dział B, Nauki Biol., (Ser. 3) 7B(47B): 193. 1907 [Jan.–Febr. 1908]	
**79**	***Silene berdaui***	Zapał.	*Silene berdaui* Zapał., Bull. Acad. Cracovie 1911. B, 286; Consp. Fl. Galic. Crit. iii. 182 (1911).	yes	Bull. Int. Acad. Sci. Cracovie, Cl. Sci. Math., Ser. B, Sci. Nat. 1911(5B): 286. 1911	
**80**	***Silene jundzillii***	Zapał.	*Silene jundzillii* Zapał., Bull. Acad. Cracovie 1911, B, 287; Consp. Fl. Galic. Crit. iii. 197.	yes	Bull. Int. Acad. Sci. Cracovie, Cl. Sci. Math., Ser. B, Sci. Nat. 1911(5B): 287. 1911	
**81**	***Silene lituanica***	Zapał.	*Silene lituanica* Zapał., Bull. Acad. Cracovie 1911, B, 285; Consp. Fl. Galic. Crit. iii. 181.	yes	Bull. Int. Acad. Sci. Cracovie, Cl. Sci. Math., Ser. B, Sci. Nat. 1911(5B): 285. 1911	
**82**	***Silene subleopoliensis***	Zapał.	*Silene subleopoliensis* Zapał., Bull. Acad. Cracovie 1911. B, 286; Consp. Fl. Galic. Crit. iii. 183.	yes	Bull. Int. Acad. Sci. Cracovie, Cl. Sci. Math., Ser. B, Sci. Nat. 1911(5B): 286. 1911	
**83**	***Sisymbrium roxolanicum***	Zapał.	*Sisymbrium roxolanicum* Zapał., Bull. Acad. Cracovie 1913, B, 48.		Bull. Int. Acad. Sci. Cracovie, Cl. Sci. Math., Ser. B, Sci. Nat. 1913(2B): 48. 1913	
**84**	***Thalictrum × andrzejowskii***	Zapał.	*Thalictrum andrzejowskii* Zapał., Consp. Fl. Galic. Crit. ii. 297 (1908).	yes	Bull. Int. Acad. Sci. Cracovie, Cl. Sci. Math. 1908(5): 450. 1908	
**85**	***Thlaspi tatrense***	Zapał.	*Thlaspi tatrense* Zapał., Bull. Acad. Cracovie 1913, B. 431; Just’s Bot. Jahresb. xli. II. 176.	yes	Bull. Int. Acad. Sci. Cracovie, Cl. Sci. Math., Ser. B, Sci. Nat. 1913(7B): 443. 1913	
**86**	***Thlaspi trojagense***	Zapał.	*Thlaspi trojagense* Zapał., Bull. Acad. Cracovie 1913, B. 444; Just’s Bot. Jahresb. xli. II. 176.	yes	Bull. Int. Acad. Sci. Cracovie, Cl. Sci. Math., Ser. B, Sci. Nat. 1913(7B): 444. 1913	
**87**	***Trisetum tarnowskii***	Zapał.	*Trisetum tarnowskii* Zapał., Bull. Acad. Cracovie 1904, 167.		Bull. Int. Acad. Sci. Cracovie, Cl. Sci. Math. 1904(3): 167. 1904	
			*Trisetum tarnowskii* Zapał., Consp. Fl. Galic. Crit. i. 35 (1906).	yes		Duplicate entry to be deleted.
**88**	***Tulipa bessarabica***	Zapał.	*Tulipa bessarabica* Zapał., Consp. Fl. Galic. Crit. i. 167 (1906).	yes	Bull. Int. Acad. Sci. Cracovie, Cl. Sci. Math. 1906(2): 101. 1906	
**89**	***Viola × babiogorensis***	Zapał.	*Viola babiogorensis* Zapał., Bull. Acad. Cracovie 1914 B. 461, hybr.		Bull. Int. Acad. Sci. Cracovie, Cl. Sci. Math., Ser. B, Sci. Nat. 1914(4B): 461. 1914	
**90**	***Viola × berdaui***	Zapał.	[absent]		Rozpr. Wydz. Mat.-Przyr. Akad. Umiejętn., Dział B, Nauki Biol., (Ser. 3) 14B(1)(54B(1)): 235. 1914 [Oct.–Dec. 1914]	
**91**	***Viola × bessarabica***	Zapał.	*Viola bessarabica* Zapał., Bull. Acad. Cracovie 1914 B. 459, hybr.		Bull. Int. Acad. Sci. Cracovie, Cl. Sci. Math., Ser. B, Sci. Nat. 1914(4B): 459. 1914	
**92**	***Viola decorata***	Zapał.	*Viola decorata* Zapał. ex Zablocki, in Rosl. Polsk., Pl. Polon. Exsicc. Ser. II. Cent. II. 13 (1934), in obs., pro syn.	yes	Rozpr. Wydz. Mat.-Przyr. Akad. Umiejętn., Dział B, Nauki Biol., (Ser. 3) 14B(1)(54B(1)): 258. 1914 [Oct.–Dec. 1914]	Authority citation in IPNI should be corrected. This name should be attributed to Zapałowicz and recorded as *Viola decorata* Zapał.
**93**	***Viola jagellonica***	Zapał.	*Viola jagellonica* Zapał., Bull. Acad. Cracovie 1914 B. 455.		Bull. Int. Acad. Sci. Cracovie, Cl. Sci. Math., Ser. B, Sci. Nat. 1914(4B): 455. 1914	
**94**	***Viola × mielnicensis***	Zapał.	*Viola mielnicensis* Zapał., Bull. Acad. Cracovie 1914 B. 463, hybr.		Bull. Int. Acad. Sci. Cracovie, Cl. Sci. Math., Ser. B, Sci. Nat. 1914(4B): 463. 1914	
**95**	***Viola × mira***	Zapał.	*Viola mira* Zapał., Bull. Acad. Cracovie 1914 B. 460, hybr.		Bull. Int. Acad. Sci. Cracovie, Cl. Sci. Math., Ser. B, Sci. Nat. 1914(4B): 460. 1914	
**96**	***Viola × prutensis***	Zapał.	*Viola prutensis* Zapał., Bull. Acad. Cracovie 1914 B. 464, hybr.		Bull. Int. Acad. Sci. Cracovie, Cl. Sci. Math., Ser. B, Sci. Nat. 1914(4B): 464. 1914	
**97**	***Viola × sanensis***	Zapał.	*Viola sanensis* Zapał., Bull. Acad. Cracovie 1914 B. 462, hybr.		Bull. Int. Acad. Sci. Cracovie, Cl. Sci. Math., Ser. B, Sci. Nat. 1914(4B): 462. 1914	
**98**	***Viola × sokalensis***	Zapał.	*Viola sokalensis* Zapał., Bull. Acad. Cracovie 1914 B. 460, hybr.		Bull. Int. Acad. Sci. Cracovie, Cl. Sci. Math., Ser. B, Sci. Nat. 1914(4B): 460. 1914	
**99**	***Viola zarencznyi***	Zapał.	*Viola zarencznyi* Zapał., Bull. Acad. Cracovie 1914 B. 457.		Bull. Int. Acad. Sci. Cracovie, Cl. Sci. Math., Ser. B, Sci. Nat. 1914(4B): 457. 1914	
**The IPNI entry incorrectly attributed to Zapałowicz**						
**1**	***Viola roxolanica***		*Viola roxolanica* Zapał., Bull. Acad. Cracovie 1914 B. 458, hybr.	yes		Entry to be deleted.
	***Viola roxolanica***	Błocki	*Viola roxolanica* Błocki, Deutsche Bot. Monatsschr. v. (1887) 147; et in Oest. Bot. Zeitschr. xxxviii.(1888) 15.			In accordance with Art. 50.1 of ICN when a taxon at the rank of species or below is transferred from the non-hybrid category to the hybrid category at the same rank (Art. H.10 Note 1), or vice versa, the authorship remains unchanged. This name is attributed to Błocki (Deutsche Bot. Monatsschr. 5: 147. 1887) and should be recorded as *Viola × roxolanica* Błocki (pro sp.).

## Appendix I

**A list of 65 publication events related to Zapałowicz’s *Conspectus florae Galiciae criticus – Krytyczny przegląd roślinności Galicyi* taking into account the division into three sources. Dates of effective publication for nomenclatural purposes are recorded in square brackets.**

Excerpt series in the *Bulletin*

1. Zapałowicz H (1904 [21 Apr. 1904]) Uwagi krytyczne nad roślinnością Galicyi. – Remarques critiques sur la flore de la Galicie. Bulletin International de l’Académie des Sciences de Cracovie. Classe des Sciences Mathématiques et Naturelles 1904(3): 162–169 (in French and Latin).

2. Zapałowicz H (1904 [5 Jul. 1904]) Krytyczny przegląd roślinności Galicyi. Część II. – Revue critique de la flore de la Galiciae (II partie). Bulletin International de l’Académie des Sciences de Cracovie. Classe des Sciences Mathématiques et Naturelles 1904(6): 302–307 (in French and Latin).

3. Zapałowicz H (1904 [15 Nov. 1904]) Krytyczny przegląd roślinności Galicyi. Część III. – Revue critique de la flore de la Galiciae (III partie). Bulletin International de l’Académie des Sciences de Cracovie. Classe des Sciences Mathématiques et Naturelles 1904(8): 394–395 (in French and Latin).

4. Zapałowicz H (1905 [16 Jun. 1905]) Krytyczny przegląd roślinności Galicyi. Część IV. – Revue critique de la flore de la Galiciae (IV partie). Bulletin International de l’Académie des Sciences de Cracovie. Classe des Sciences Mathématiques et Naturelles 1905(5): 286 (in French and Latin).

5. Zapałowicz H (1906 [15 Mar. 1906]) Krytyczny przegląd roślinności Galicyi. Część V. – Revue critique de la flore de la Galiciae (V partie). Bulletin International de l’Académie des Sciences de Cracovie. Classe des Sciences Mathématiques et Naturelles 1906(2): 100–101 (in French).

6. Zapałowicz H (1906 [25 Jun. 1906]) Krytyczny przegląd roślinności Galicyi. Część VI. – Revue critique de la flore de la Galiciae (VI partie). Bulletin International de l’Académie des Sciences de Cracovie. Classe des Sciences Mathématiques et Naturelles 1906(5): 326–327 (in French and Latin).

7. Zapałowicz H (1906 [19 Oct. 1906]) Krytyczny przegląd roślinności Galicyi. Część VII. – Revue critique de la flore de la Galiciae (VII partie). Bulletin International de l’Académie des Sciences de Cracovie. Classe des Sciences Mathématiques et Naturelles 1906(7): 603 (in French).

8. Zapałowicz H (1907 [12 Mar. 1907]) Krytyczny przegląd roślinności Galicyi. Część VIII. – Revue critique de la flore de la Galiciae (VIII partie). Bulletin International de l’Académie des Sciences de Cracovie. Classe des Sciences Mathématiques et Naturelles 1907(2): 59–60 (in French and Latin).

9. Zapałowicz H (1907 [15 May 1907]) Krytyczny przegląd roślinności Galicyi. Część IX. – Revue critique de la flore de la Galiciae (IX partie). Bulletin International de l’Académie des Sciences de Cracovie. Classe des Sciences Mathématiques et Naturelles 1907(4): 253–254 (in French and Latin).

10. Zapałowicz H (1907 [10 Aug. 1907]) Krytyczny przegląd roślinności Galicyi. Część X. – Revue critique de la flore de la Galiciae (X partie). Bulletin International de l’Académie des Sciences de Cracovie. Classe des Sciences Mathématiques et Naturelles 1907(6): 631–632 (in French and Latin).

11. Zapałowicz H (1907 [28 Dec. 1907]) Krytyczny przegląd roślinności Galicyi. Część XI. – Revue critique de la flore de la Galiciae (XI partie). Bulletin International de l’Académie des Sciences de Cracovie. Classe des Sciences Mathématiques et Naturelles 1907(10): 1079–1080 (in French and Latin).

12. Zapałowicz H (1908 [12 Mar. 1908]) Krytyczny przegląd roślinności Galicyi. Część XII. – Revue critique de la flore de la Galiciae (XII partie). Bulletin International de l’Académie des Sciences de Cracovie. Classe des Sciences Mathématiques et Naturelles 1908(3): 141–145 (in French and Latin).

13. Zapałowicz H (1908 [3 Jun. 1908]) Krytyczny przegląd roślinności Galicyi. Część XIII. – Revue critique de la flore de la Galiciae (XIII partie). Bulletin International de l’Académie des Sciences de Cracovie. Classe des Sciences Mathématiques et Naturelles 1908(5): 448–450 (in French and Latin).

14. Zapałowicz H (1908 [14 Sept. 1908]) Krytyczny przegląd roślinności Galicyi. Część XIV. – Revue critique de la flore de la Galiciae (XIV partie). Bulletin International de l’Académie des Sciences de Cracovie. Classe des Sciences Mathématiques et Naturelles 1908(7): 603 (in French and Latin).

15. Zapałowicz H (1910 [12 Apr. 1910]) Krytyczny przegląd roślinności Galicyi. Część XV. – Revue critique de la flore de la Galiciae (XV partie). Bulletin International de l’Académie des Sciences de Cracovie. Classe des Sciences Mathématiques et Naturelles. Série B. Sciences Naturelles 1910(3B): 168–172 (in French).

16. Zapałowicz H (1910 [29 Jul. 1910]) Krytyczny przegląd roślinności Galicyi. Część XVI. – Revue critique de la flore de la Galiciae (XVI partie). Bulletin International de l’Académie des Sciences de Cracovie. Classe des Sciences Mathématiques et Naturelles. Série B. Sciences Naturelles 1910(6B): 433–438 (in French and Latin).

17. Zapałowicz H (1910 [10 Sep. 1910]) Krytyczny przegląd roślinności Galicyi. Część XVII. – Revue critique de la flore de la Galiciae (XVII partie). Bulletin International de l’Académie des Sciences de Cracovie. Classe des Sciences Mathématiques et Naturelles. Série B. Sciences Naturelles 1910(7B): 607 (in French).

18. Zapałowicz H (1911 [9 Feb. 1911]) Krytyczny przegląd roślinności Galicyi. Część XVIII. – Revue critique de la flore de la Galiciae (XVIII partie). Bulletin International de l’Académie des Sciences de Cracovie. Classe des Sciences Mathématiques et Naturelles. Série B. Sciences Naturelles 1911(1B): 7–11 (in French and Latin).

19. Zapałowicz H (1911 [12 Apr. 1911]) Krytyczny przegląd roślinności Galicyi. Część XIX. – Revue critique de la flore de la Galiciae (XIX partie). Bulletin International de l’Académie des Sciences de Cracovie. Classe des Sciences Mathématiques et Naturelles. Série B. Sciences Naturelles 1911(3B): 162–163 (in French and Latin).

20. Zapałowicz H (1911 [26 Jun. 1911]) Krytyczny przegląd roślinności Galicyi. Część XX. – Revue critique de la flore de la Galiciae (XX partie). Bulletin International de l’Académie des Sciences de Cracovie. Classe des Sciences Mathématiques et Naturelles. Série B. Sciences Naturelles 1911(5B): 285–289 (in French and Latin).

21. Zapałowicz H (1911 [27 Jul. 1911]) Krytyczny przegląd roślinności Galicyi. Część XXI. – Revue critique de la flore de la Galiciae (XXI partie). Bulletin International de l’Académie des Sciences de Cracovie. Classe des Sciences Mathématiques et Naturelles. Série B. Sciences Naturelles 1911(6B): 497–499 (in French and Latin).

22. Zapałowicz H (1911 [25 Nov. 1911]) Krytyczny przegląd roślinności Galicyi. Część XXII. – Revue critique de la flore de la Galiciae (XXII partie). Bulletin International de l’Académie des Sciences de Cracovie. Classe des Sciences Mathématiques et Naturelles. Série B. Sciences Naturelles 1911(8B): 620–622 (in French and Latin).

23. Zapałowicz H (1912 [26 Feb. 1912]) Krytyczny przegląd roślinności Galicyi. Część XXIII. – Revue critique de la flore de la Galiciae (XXIII partie) [1^st^ part]. Bulletin International de l’Académie des Sciences de Cracovie. Classe des Sciences Mathématiques et Naturelles. Série B. Sciences Naturelles 1912(1B): 12–16 (in French and Latin).

24. Zapałowicz H (1912 [20 Mar. 1912]) Krytyczny przegląd roślinności Galicyi. Część XXIII. – Revue critique de la flore de la Galiciae (XXIII partie) [2^nd^ part]. Bulletin International de l’Académie des Sciences de Cracovie. Classe des Sciences Mathématiques et Naturelles. Série B. Sciences Naturelles 1912(2B): 17–22 (in Latin).

25. Zapałowicz H (1912 [13 Jun. 1912]) Krytyczny przegląd roślinności Galicyi. Część XXIV. – Revue critique de la flore de la Galiciae (XXIV partie). Bulletin International de l’Académie des Sciences de Cracovie. Classe des Sciences Mathématiques et Naturelles. Série B. Sciences Naturelles 1912(4B): 345–348 (in French and Latin).

26. Zapałowicz H (1912 [9 Nov. 1912]) Krytyczny przegląd roślinności Galicyi. Część XXV. – Revue critique de la flore de la Galiciae (XXV partie). Bulletin International de l’Académie des Sciences de Cracovie. Classe des Sciences Mathématiques et Naturelles. Série B. Sciences Naturelles 1912(7B): 710–716 (in French and Latin).

27. Zapałowicz H (1913 [12 Jan. 1913]) Krytyczny przegląd roślinności Galicyi. Część XXVI. – Revue critique de la flore de la Galiciae (XXVI partie). Bulletin International de l’Académie des Sciences de Cracovie. Classe des Sciences Mathématiques et Naturelles. Série B. Sciences Naturelles 1912(9B): 1158 (in French and Latin).

28. Zapałowicz H (1913 [15 Mar. 1913]) Krytyczny przegląd roślinności Galicyi. Część XXVII. – Revue critique de la flore de la Galiciae (XXVII partie) [1^st^ part]. Bulletin International de l’Académie des Sciences de Cracovie. Classe des Sciences Mathématiques et Naturelles. Série B. Sciences Naturelles 1913(2B): 48 (in French and Latin).

29. Zapałowicz H (1913 [24 Apr. 1913]) Krytyczny przegląd roślinności Galicyi. Część XXVII. – Revue critique de la flore de la Galiciae (XXVII partie) [2^nd^ part]. Bulletin International de l’Académie des Sciences de Cracovie. Classe des Sciences Mathématiques et Naturelles. Série B. Sciences Naturelles 1913(3B): 49–50 (in Latin).

30. Zapałowicz H (1913 [30 Jun. 1913]) Krytyczny przegląd roślinności Galicyi. Część XXVIII. – Revue critique de la flore de la Galiciae (XXVIII partie). Bulletin International de l’Académie des Sciences de Cracovie. Classe des Sciences Mathématiques et Naturelles. Série B. Sciences Naturelles 1913(5B): 273–274 (in French and Latin).

31. Zapałowicz H (1913 [10 Oct. 1913]) Krytyczny przegląd roślinności Galicyi. Część XXIX. – Revue critique de la flore de la Galiciae (XXIX partie). Bulletin International de l’Académie des Sciences de Cracovie. Classe des Sciences Mathématiques et Naturelles. Série B. Sciences Naturelles 1913(7B): 443–448 (in French and Latin).

32. Zapałowicz H (1914 [23 May 1914]) Krytyczny przegląd roślinności Galicyi. Część XXX. – Revue critique de la flore de la Galiciae (XXX partie). Bulletin International de l’Académie des Sciences de Cracovie. Classe des Sciences Mathématiques et Naturelles. Série B. Sciences Naturelles 1914(4B): 455–464 (in French and Latin).

Serial form in the *Rozprawy*

33. Zapałowicz H (1904 [Aug.–Oct. 1905]) Conspectus florae Galiciae criticus [pars I]. – Krytyczny przegląd roślinności Galicyi [część I]. Rozprawy Wydziału Matematyczno-Przyrodniczego Akademii Umiejętności, Dział B. Nauki Biologiczne (Seria 3) 4B(44B): 74–113 (in Polish and Latin).

34. Zapałowicz H (1904 [Aug.–Oct. 1905]) Conspectus florae Galiciae criticus (pars II). – Krytyczny przegląd roślinności Galicyi (część II). Rozprawy Wydziału Matematyczno-Przyrodniczego Akademii Umiejętności, Dział B. Nauki Biologiczne (Seria 3) 4B(44B): 153–196 (in Polish and Latin).

35. Zapałowicz H (1904 [Aug.–Oct. 1905]) Conspectus florae Galiciae criticus (pars III). – Krytyczny przegląd roślinności Galicyi (część III). Rozprawy Wydziału Matematyczno-Przyrodniczego Akademii Umiejętności, Dział B. Nauki Biologiczne (Seria 3) 4B(44B): 305–341 (in Polish and Latin).

36. Zapałowicz H (1906 [Aug.–Oct. 1906]) Conspectus florae Galiciae criticus (pars IV). – Krytyczny przegląd roślinności Galicyi (część IV). Rozprawy Wydziału Matematyczno-Przyrodniczego Akademii Umiejętności, Dział B. Nauki Biologiczne (Seria 3) 5B(45B): 83–110 (in Polish and Latin).

37. Zapałowicz H (1906 [Dec. 1906]) Conspectus florae Galiciae criticus (pars V). – Krytyczny przegląd roślinności Galicyi (część V). Rozprawy Wydziału Matematyczno-Przyrodniczego Akademii Umiejętności, Dział B. Nauki Biologiczne (Seria 3) 6B(46B): 65–102 (in Polish and Latin).

38. Zapałowicz H (1906 [Dec. 1906]) Conspectus florae Galiciae criticus (pars VI). – Krytyczny przegląd roślinności Galicyi (część VI). Rozprawy Wydziału Matematyczno-Przyrodniczego Akademii Umiejętności, Dział B. Nauki Biologiczne (Seria 3) 6B(46B): 189–239 (in Polish and Latin).

39. Zapałowicz H (1906 [Dec. 1906 (pp. 241–288) / Mar. 1907 (pp. 289–296)]) Conspectus florae Galiciae criticus (pars VII). – Krytyczny przegląd roślinności Galicyi (część VII). Rozprawy Wydziału Matematyczno-Przyrodniczego Akademii Umiejętności, Dział B. Nauki Biologiczne (Seria 3) 6B(46B): 241–296 (in Polish and Latin).

40. Zapałowicz H (1907 [Jan.–Febr. 1908]) Conspectus florae Galiciae criticus (pars VIII). – Krytyczny przegląd roślinności Galicyi (część VIII). Rozprawy Wydziału Matematyczno-Przyrodniczego Akademii Umiejętności, Dział B. Nauki Biologiczne (Seria 3) 7B(47B): 153–236 (in Polish and Latin).

41. Zapałowicz H (1907 [Jan.–Febr. 1908]) Conspectus florae Galiciae criticus (pars IX). – Krytyczny przegląd roślinności Galicyi (część IX). Rozprawy Wydziału Matematyczno-Przyrodniczego Akademii Umiejętności, Dział B. Nauki Biologiczne (Seria 3) 7B(47B): 265–302 (in Polish and Latin).

42. Zapałowicz H (1907 [Jan.–Febr. 1908]) Conspectus florae Galiciae criticus (pars X). – Krytyczny przegląd roślinności Galicyi (część X). Rozprawy Wydziału Matematyczno-Przyrodniczego Akademii Umiejętności, Dział B. Nauki Biologiczne (Seria 3) 7B(47B): 587–631 (in Polish and Latin).

43. Zapałowicz H (1907 [Mar. 1908]) Conspectus florae Galiciae criticus (pars XI). – Krytyczny przegląd roślinności Galicyi (część XI). Rozprawy Wydziału Matematyczno-Przyrodniczego Akademii Umiejętności, Dział B. Nauki Biologiczne (Seria 3) 7B(47B): 685–703 (in Polish and Latin).

44. Zapałowicz H (1909 [Jul.–Aug. 1908]) Conspectus florae Galiciae criticus (pars XII). – Krytyczny przegląd roślinności Galicyi (część XII). Rozprawy Wydziału Matematyczno-Przyrodniczego Akademii Umiejętności, Dział B. Nauki Biologiczne (Seria 3) 8B(48B): 41–90 (in Polish and Latin).

45. Zapałowicz H (1909 [Jul.–Aug. 1908]) Conspectus florae Galiciae criticus (pars XIII). – Krytyczny przegląd roślinności Galicyi (część XIII). Rozprawy Wydziału Matematyczno-Przyrodniczego Akademii Umiejętności, Dział B. Nauki Biologiczne (Seria 3) 8B(48B): 187–256 (in Polish and Latin).

46. Zapałowicz H (1909 [Apr.–May 1909]) Conspectus florae Galiciae criticus (pars XIV). – Krytyczny przegląd roślinności Galicyi (część XIV). Rozprawy Wydziału Matematyczno-Przyrodniczego Akademii Umiejętności, Dział B. Nauki Biologiczne (Seria 3) 8B(48B): 331–336 (in Polish and Latin).

47. Zapałowicz H (1911 [Jan. 1911]) Conspectus florae Galiciae criticus (pars XV). – Krytyczny przegląd roślinności Galicyi (część XV). Rozprawy Wydziału Matematyczno-Przyrodniczego Akademii Umiejętności, Dział B. Nauki Biologiczne (Seria 3) 10B(50B): 173–211 (in Polish and Latin).

48. Zapałowicz H (1911 [Jan. 1911]) Conspectus florae Galiciae criticus (pars XVI). – Krytyczny przegląd roślinności Galicyi (część XVI). Rozprawy Wydziału Matematyczno-Przyrodniczego Akademii Umiejętności, Dział B. Nauki Biologiczne (Seria 3) 10B(50B): 353–408 (in Polish and Latin).

49. Zapałowicz H (1911 [Jan. 1911]) Conspectus florae Galiciae criticus (pars XVII). – Krytyczny przegląd roślinności Galicyi (część XVII). Rozprawy Wydziału Matematyczno-Przyrodniczego Akademii Umiejętności, Dział B. Nauki Biologiczne (Seria 3) 10B(50B): 671–682 (in Polish and Latin).

50. Zapałowicz H (1911 [Mar. 1912]) Conspectus florae Galiciae criticus (pars XVIII). – Krytyczny przegląd roślinności Galicyi (część XVIII). Rozprawy Wydziału Matematyczno-Przyrodniczego Akademii Umiejętności, Dział B. Nauki Biologiczne (Seria 3) 11B(51B): 1–34 (in Polish and Latin).

51. Zapałowicz H (1911 [Mar. 1912]) Conspectus florae Galiciae criticus (pars XIX). – Krytyczny przegląd roślinności Galicyi (część XIX). Rozprawy Wydziału Matematyczno-Przyrodniczego Akademii Umiejętności, Dział B. Nauki Biologiczne (Seria 3) 11B(51B): 67–89 (in Polish and Latin).

52. Zapałowicz H (1911 [Mar. 1912]) Conspectus florae Galiciae criticus (pars XX). – Krytyczny przegląd roślinności Galicyi (część XX). Rozprawy Wydziału Matematyczno-Przyrodniczego Akademii Umiejętności, Dział B. Nauki Biologiczne (Seria 3) 11B(51B): 121–157 (in Polish and Latin).

53. Zapałowicz H (1911 [Mar. 1912]) Conspectus florae Galiciae criticus (pars XXI). – Krytyczny przegląd roślinności Galicyi (część XXI). Rozprawy Wydziału Matematyczno-Przyrodniczego Akademii Umiejętności, Dział B. Nauki Biologiczne (Seria 3) 11B(51B): 265–280 (in Polish and Latin).

54. Zapałowicz H (1911 [Mar. 1912]) Conspectus florae Galiciae criticus (pars XXII). – Krytyczny przegląd roślinności Galicyi (część XXII). Rozprawy Wydziału Matematyczno-Przyrodniczego Akademii Umiejętności, Dział B. Nauki Biologiczne (Seria 3) 11B(51B): 443–456 (in Polish and Latin).

55. Zapałowicz H (1912 [Feb. 1913]) Conspectus florae Galiciae criticus (pars XXIII). – Krytyczny przegląd roślinności Galicyi (część XXIII). Rozprawy Wydziału Matematyczno-Przyrodniczego Akademii Umiejętności, Dział B. Nauki Biologiczne (Seria 3) 12B(52B): 1–49 (in Polish and Latin).

56. Zapałowicz H (1912 [Feb. 1913]) Conspectus florae Galiciae criticus (pars XXIV). – Krytyczny przegląd roślinności Galicyi (część XXIV). Rozprawy Wydziału Matematyczno-Przyrodniczego Akademii Umiejętności, Dział B. Nauki Biologiczne (Seria 3) 12B(52B): 157–180 (in Polish and Latin).

57. Zapałowicz H (1912 [Feb. 1913]) Conspectus florae Galiciae criticus (pars XXV). – Krytyczny przegląd roślinności Galicyi (część XXV). Rozprawy Wydziału Matematyczno-Przyrodniczego Akademii Umiejętności, Dział B. Nauki Biologiczne (Seria 3) 12B(52B): 211–239 (in Polish and Latin).

58. Zapałowicz H (1912 [Feb. 1913]) Conspectus florae Galiciae criticus (pars XXVI). – Krytyczny przegląd roślinności Galicyi (część XXVI). Rozprawy Wydziału Matematyczno-Przyrodniczego Akademii Umiejętności, Dział B. Nauki Biologiczne (Seria 3) 12B(52B): 547–565 (in Polish and Latin).

59. Zapałowicz H (1913 [Apr. 1914]) Conspectus florae Galiciae criticus (pars XXVII). – Krytyczny przegląd roślinności Galicyi (część XXVII). Rozprawy Wydziału Matematyczno-Przyrodniczego Akademii Umiejętności, Dział B. Nauki Biologiczne (Seria 3) 13B(53B): 29–49 (in Polish and Latin).

60. Zapałowicz H (1913 [Apr. 1914]) Conspectus florae Galiciae criticus (pars XXVIII). – Krytyczny przegląd roślinności Galicyi (część XXVIII). Rozprawy Wydziału Matematyczno-Przyrodniczego Akademii Umiejętności, Dział B. Nauki Biologiczne (Seria 3) 13B(53B): 115–125 (in Polish and Latin).

61. Zapałowicz H (1913 [Apr. 1914]) Conspectus florae Galiciae criticus (pars XXIX). – Krytyczny przegląd roślinności Galicyi (część XXIX). Rozprawy Wydziału Matematyczno-Przyrodniczego Akademii Umiejętności, Dział B. Nauki Biologiczne (Seria 3) 13B(53B): 311–339 (in Polish and Latin).

62. Zapałowicz H (1914 [Oct.–Dec. 1914]) Conspectus florae Galiciae criticus (pars XXX). – Krytyczny przegląd roślinności Galicyi (część XXX). Rozprawy Wydziału Matematyczno-Przyrodniczego Akademii Umiejętności, Dział B. Nauki Biologiczne (Seria 3) 14B(1)(54(1)): 209–270 (in Polish and Latin).

Book form

63. Zapałowicz H (1906 [Aug.–Oct. 1906]) Conspectus florae Galiciae criticus, Vol. 1. – Krytyczny przegląd roślinności Galicyi, Tom 1. Akademia Umiejętności, Kraków, 1–296 (in Polish and Latin).

64. Zapałowicz H (1908 [Aug.–Oct. 1908]) Conspectus florae Galiciae criticus, Vol. 2. – Krytyczny przegląd roślinności Galicyi, Tom 2. Akademia Umiejętności, Kraków, 1–311 (in Polish and Latin).

65. Zapałowicz H (1911 [Nov. 1911]) Conspectus florae Galiciae criticus, Vol. 3. – Krytyczny przegląd roślinności Galicyi, Tom 3. Akademia Umiejętności, Kraków, 1−246 (in Polish and Latin).

## Supplementary materials

Supplementary material 1 Table S1 Data type bibliographic data, species data Explanation note Bibliographic details of the 30 excerpts of Zapałowicz’s series *Krytyczny przegląd roślinności Galicyi* – *Revue critique de la flore de la Galicie published in Bulletin International de l’Académie des Sciences de Cracovie*. https://binary.pensoft.net/file/440103This dataset is made available under the Open Database License (http://opendatacommons.org/licenses/odbl/1.0/). The Open Database License (ODbL) is a license agreement intended to allow users to freely share, modify, and use this Dataset while maintaining this same freedom for others, provided that the original source and author(s) are credited.Beata Paszko, Agnieszka Nikel, Aldona Mueller-Bieniek, Wojciech Paul

Supplementary material 2 Table S2 Data type bibliographic data Explanation note Bibliographic details of the 30 individual parts of Zapałowicz’s series *Conspectus florae Galiciae criticus*. – *Krytyczny przegląd roślinności Galicyi published in the Rozprawy Wydziału Matematyczno*-*Przyrodniczego Akademii Umiejętności*, *Dział B. Nauki Biologiczne* (*Seria 3*). https://binary.pensoft.net/file/440104This dataset is made available under the Open Database License (http://opendatacommons.org/licenses/odbl/1.0/). The Open Database License (ODbL) is a license agreement intended to allow users to freely share, modify, and use this Dataset while maintaining this same freedom for others, provided that the original source and author(s) are credited.Beata Paszko, Agnieszka Nikel, Aldona Mueller-Bieniek, Wojciech Paul

Supplementary material 3 Table S3 Data type bibliographic data Explanation note Receipts for the Academy of Arts and Sciences provided by the Jagiellonian University Printing House for printing subsequent signatures of the *Rozprawy Wydziału Matematyczno*-*Przyrodniczego Akademii Umiejętności*, *Dział B. Nauki Biologiczne* (*Seria 3*). https://binary.pensoft.net/file/440105This dataset is made available under the Open Database License (http://opendatacommons.org/licenses/odbl/1.0/). The Open Database License (ODbL) is a license agreement intended to allow users to freely share, modify, and use this Dataset while maintaining this same freedom for others, provided that the original source and author(s) are credited.Beata Paszko, Agnieszka Nikel, Aldona Mueller-Bieniek, Wojciech Paul

Supplementary material 4 Table S4 Data type bibliographic data Explanation note Bibliographic details of Zapałowicz’s *Conspectus florae Galiciae criticus* – *Krytyczny przegląd roślinności Galicyi* (3 Vols). https://binary.pensoft.net/file/440106This dataset is made available under the Open Database License (http://opendatacommons.org/licenses/odbl/1.0/). The Open Database License (ODbL) is a license agreement intended to allow users to freely share, modify, and use this Dataset while maintaining this same freedom for others, provided that the original source and author(s) are credited.Beata Paszko, Agnieszka Nikel, Aldona Mueller-Bieniek, Wojciech Paul
