# Supplementary material for: Zapałowicz’s Conspectus florae Galiciae criticus: Clarification of publication dates for nomenclatural purposes and bibliographic notes
Source: PhytoKeys. 2020 Aug 7;155:53–85. doi: 10.3897/phytokeys.155.51072 (PMC7443691; doi:10.3897/phytokeys.155.51072)
Supplement: Supplementary material 1 — Table S1 [file phytokeys-155-053-s001.pdf]

Supplementary material 1. Bibliographic details of the 30 excerpts of Zapałowicz's series titled *Krytyczny przegląd roślinności Galicyi – Revue critique de la flore de la Galicie* published in the *Bulletin International de l'Académie des Sciences de Cracovie. Classe des Sciences Mathématiques et Naturelles*, and in its successor, *Bulletin International de l'Académie des Sciences de Cracovie. Classe des Sciences Mathématiques et Naturelles. Série B. Sciences Naturelles* (the *Bulletin*). Abbreviations: AAS – the Academy of Arts and Sciences (Kraków).

Part	Date of presentation at the AAS meeting	The *Bulletin*					Names of taxa described by Hugo Zapałowicz (species and nothospecies in bold)
		Date on the volume title page	Volume	Issue (livraison) number and year on its front wrapper	Pages	Issue's publication date	
[1]	7 Mar. 1904	1905	1904	3, 1904	162–169	21 Apr. 1904	*Phleum alpinum* L. var. *elongatum* ["*elongata*"] Zapał., *Agrostis alba* L. var. *pauciflora* Zapał., *A. canina* L. var. *breviaristata* Zapał., *A. rupestris* All. var. *subscabra* Zapał., ***Calamagrostis kotulae*** Zapał., *C. villosa* Mutel var. *krupae* Zapał., *Avena elatior* var. *carpatica* Zapał., *A. pubescens* Huds. var. *minor* Zapał., *A. pratensis* L. [var.] *scabra* Zapał., [var.] *glabrata* Zapał., *A. planiculmis* Schrad. [var.] *czywczynensis* Zapał., [var.] *hispidula* Zapał., *Trisetum flavescens* P. Beauv. var. *paczoskii* Zapał. (incl. f. *scabriuscula* Zapał., f. *subpilosa* Zapał.), ***T. tarnowskii*** Zapał., *T. alpestre* P. Beauv. (incl. [var.] *aurea* Zapał., [var.] *tatrensis* Zapał.)
2	6 Jun. 1904			6, 1904	302–307	5 Jul. 1904	***Festuca polonica*** Zapał., ***F. polesica*** Zapał., ***F. pietrosii*** Zapał., ***Bromus janczewskii*** Zapał.
3	17 Oct. 1904			8, 1904	394–395	15 Nov. 1904	–
4	9 May 1905	1906	1905	5, 1905	286	16 Jun. 1905	–
5	5 Feb. 1906	1907	1906	2, 1906	100–101	15 Mar. 1906	***Muscari pocuticum*** Zapał., ***Tulipa bessarabica*** Zapał.
6	7 May 1906			5, 1906	326–327	25 Jun. 1906	***Crocus babiogorensis*** Zapał., ***Iris pontica*** Zapał.
7	7 May 1906			7, 1906	603	19 Oct. 1906	–
8	4 Feb. 1907	1907	1907	2, 1907	59–60	12 Mar. 1907	***Salix tatrorum*** Zapał.
9	9 Apr. 1907			4, 1907	253–254	15 May 1907	***Rumex carpaticus*** Zapał., ***R. ×babiogorensis*** Zapał.
10	3 Jun. 1907			6, 1907	631–632	10 Aug. 1907	***Polygonum ×janoviense*** Zapał., ***P. ×asperulum*** Zapał.
11	2 Dec. 1907			10, 1907	1079–1080	28 Dec. 1907	***Atriplex polonicum*** Zapał.
12	2 Mar. 1908	1909	1908	3, 1908	141–145	12 Mar. 1908	***Delphinium nacladense*** Zapał. (incl. f. *elegans* Zapał., var. *pietrosuanum* Zapał.), ***Aconitum ×berdaui*** Zapał., ***A. ×bucovinense*** Zapał.
13	4 May 1908			5, 1908	448–450	3 Jun. 1908	***Pulsatilla ×janczewskii*** Zapał. (incl. f. *pluriscapa* Zapał.), ***P. ×tarnoviensis*** Zapał., ***Ranunculus ×klukii*** Zapał., ***R. ×gilibertii*** Zapał., ***Thalictrum ×andrzejowskii*** Zapał.
14	22 Jun. 1908			7, 1908	603	14 Sept. 1908	–
15	7 Mar. 1910	1911	1910	3B, 1910	168–172	12 Apr. 1910	***Alsine zarencznyi*** Zapał. (incl. f. *subpurpurea* Zapał., f. *supraglandulosa* Zapał., f. *paucicaulis* Zapał., f. *minima* Zapał., f. *bryophila* Zapał., [var.] *neglecta* Zapał. (incl. f. *subcolorata* Zapał., f. *subcaespitosa* Zapał., f. *ramificans* Zapał.), [var.] *devestita* Zapał., [var.] *oxypetala* Woł. (incl. f. *acutissima* Zapał., f. *micropetala* Zapał.), [var.] *pseudogerardiana* Zapał.
16	6 Jun. 1910			6B, 1910	433–438	29 Jul. 1910	***Cerastium raciborskii*** Zapał. (incl. f. *giewonticum* Zapał., f. *bistrense* Zapał., f. *rigidulum* Zapał., f. *subglabrum* Zapał., var. *morskiense* Zapał. (incl. f. *intermedium* Zapał.)), ***C. ciarcanense*** Zapał., ***C. pietrosuanum*** Zapał., ***C. ×tatrense*** Zapał.
17	4 Jul. 1910			7B, 1910	607	10 Sep. 1910	*Gypsophila paniculata* subsp. *lithuanica* Zapał. ["*lituanica*"]
18	9 Jan. 1911	1912	1911	1B, 1911	7–11	9 Feb. 1911	***Dianthus polonicus*** Zapał., *D. capitatus* DC. subsp. *andrzejowskianus* Zapał., ***D. euponticus*** Zapał.
19	6 Mar. 1911			3B, 1911	162–163	12 Apr. 1911	***Dianthus ×zarencznianus*** Zapał., ***D. ×lacinulatus*** Zapał.
20	1 May 1911			5B, 1911	285–289	26 Jun. 1911	***Silene lithuanica*** Zapał. ["*lituanica*"], ***S. berdaui*** Zapał. (incl. f. *latiuscula* Zapał.), ***S. subleopoliensis*** Zapał., ***S. jundzillii*** Zapał. (incl. [var.] *typica* [*nom. illeg.*] (incl. f. *aucta* Zapał., f. *choczensis* Zapał., f. *sparsiflora* Zapał.), [var.] *hryniawiensis* Zapał., [var.] *pienina* Zapał. (incl. f. *subglabra* Zapał.), [var.] *brachyantha* Zapał.)
21	12 Jun. 1911			6B, 1911	497–499	27 Jul. 1911	*Heliosperma quadrifidum* (L.) Rchb. subsp. *carpaticum* Zapał. (incl. f. *laticordatum* Zapał., [var.] *grandiflorum* Zapał. (incl. f. *ineunense* Zapał.), [var.] *rodnense* Zapał.), ***H. arcanum*** Zapał.
22	9 Oct. 1911			8B, 1911	620–622	25 Nov. 1911	***Papaver corona-sancti-stephani*** Zapał. ["*corona Sti Stephani*"] (incl. f. *hispidulum* Zapał., var. *angustisectum* Zapał.)
23a	5 Feb. 1912	1913	1912	1B, 1912	12–16	26 Feb. 1912	***Cardamine ×tatrensis*** Zapał., ***C. ×dubia*** Zapał., *Arabis arenosa* (L.) Scop. subsp. *borbasii* Zapał. (incl. f. *rodnensis* Zapał., f. *ineuensis* Zapał., f. *innovans* Zapał., f. *sublongifolia* Zapał., f. *choczensis* Zapał., f. *swidoviensis* Zapał., f. *suffruticosa* Zapał., f. *babiogorensis* Zapał., [var.] *tatrensis* Zapał.)
23b	5 Feb. 1912			2B, 1912	17–22	20 Mar. 1912	*Arabis arenosa* (L.) Scop. subsp. *borbasii* Zapał. [var.] *tatrensis* Zapał. f. *platyphylla* Zapał., ***Arabis besseri*** Zapał. (incl. [var.] *typica* [*nom. illeg.*], [var.] *duriuscula* Zapał. (incl. f. *minor* Zapał.), [var.] *miodoborensis* Zapał., subsp. *proseocarpatica* Zapał.), ***A. ×decipiens*** Zapał., ***A. ×kotulae*** Zapał., ***A. ×calcigena*** Zapał., ***A. ×saccata*** Zapał.
24	1 Apr. 1912			4B, 1912	345–348	13 Jun. 1912	***Rorippa cracoviensis*** Zapał. ["*Roripa*"], ***R. ×podolica*** Zapał., ***R. ×viaria*** Zapał., ***R. ×sodalis*** Zapał., ***R. ×oslawiensis*** Zapał., ***R. ×wislokiensis*** Zapał.
25	1 Jul. 1912			7B, 1912	710–716	9 Nov. 1912	***Alyssum borysthenicum*** Zapał., ***Alyssum brodense*** Zapał., *Draba aizoides* L. subsp. *zmudae* Zapał. ((incl. f. *elatior* Zapał., f. *minima* Zapał., f. *cordigera* Zapał., f. *longiflora* Zapał., f. *latiuscula* Zapał., f. *stenocarpa* Zapał., f. *platycarpa* Zapał., f. *subvestita* Zapał.), var. *marmarosiensis* Zapał.), *Draba carinthiaca* Hoppe subsp. *orientigena* Zapał. ((incl. f. *ramificans* Zapał., f. *longiuscula* Zapał., f. *suchardensis* Zapał., f. *czarnohorensis* Zapał. (incl. subf. *adscendens* Zapał.), f. *bardovensis* Zapał.), var. *swidoviensis* Zapał.)
26	4 Nov. 1912			9B, 1913	1158	12 Jan. 1913	***Hesperis pontica*** Zapał.
27a	3 Mar. 1913	1914	1913	2B, 1913	48	15 Mar. 1913	***Sisymbrium roxolanicum*** Zapał. ["*Sisymbrum*"]
27b	3 Mar. 1913			3B, 1913	49–50	24 Apr. 1913	***Erysimum hungaricum*** Zapał. (incl. var. *subdiscolor* Zapał.)
28	5 May 1913			5B, 1913	273–274	30 Jun. 1913	***Diplotaxis polonica*** Zapał.
29	7 Jul. 1913			7B, 1913	443–448	10 Oct. 1913	***Thlaspi tatrense*** Zapał. ["*Thlapsi*"] (incl. f. *laxiusculum* Zapał.), ***T. trojagense*** Zapał. (incl. f. *abbreviatum* Zapał.), ***Bunias dubia*** Zapał., ***Isatis kamienskii*** Zapał., ***Isatis ciesielskii*** Zapał.
30	27 Apr. 1914	1914	1914	4B, 1914	455–464	23 May 1914	***Viola jagellonica*** Zapał. (incl. var. *colorata* Zapał.), ***V. zarencznyi*** Zapał. (incl. var. *micropetala* Zapał.), ***V. ×bessarabica*** Zapał., ***V. ×mira*** Zapał., ***V. ×sokalensis*** Zapał., ***V. ×babiogorensis*** Zapał. (incl. var. *mariae* Zapał.), ***V. ×sanensis*** Zapał. (incl. var. *subleopoliensis* Zapał.), ***V. ×mielnicensis*** Zapał., ***V. ×prutensis*** Zapał.
