# Supplementary material for: Zapałowicz’s Conspectus florae Galiciae criticus: Clarification of publication dates for nomenclatural purposes and bibliographic notes
Source: PhytoKeys. 2020 Aug 7;155:53–85. doi: 10.3897/phytokeys.155.51072 (PMC7443691; doi:10.3897/phytokeys.155.51072)
Supplement: Supplementary material 2 — Table S2 [file phytokeys-155-053-s002.pdf]

Supplementary material 2. Bibliographic details of the 30 individual parts of Zapałowicz's series titled *Conspectus florae Galiciae criticus – Krytyczny przegląd roślinności Galicyi* published in the *Rozprawy Wydziału Matematyczno-Przyrodniczego Akademii Umiejętności, Dział B. Nauki Biologiczne (Seria 3)* (the *Rozprawy*). Abbreviations: AAS – the Academy of Arts and Sciences (Kraków), DD – Data deficient, Gebethner's Catalogue – *Katalog Nowych Książek: miesięcznik bibliograficzny Księgarni G. Gebethnera i Spółki w Krakowie* [= Catalogue of New Books: bibliographic monthly journal of G. Gebethner & Co. Publishing House in Kraków], the *Rocznik AU – Rocznik Akademii Umiejętności w Krakowie* [= AAS Annual (Kraków)], the *Spraw. AU – Sprawozdania z Czynności i Posiedzeń Akademii Umiejętności w Krakowie* [= Reports on the AAS's Activities and Meetings (Kraków)], *Tromsø Mus. Aarsberetn. – Tromsø Museum Aarsberetning*.

Part	The *Rozprawy*					Dates of publication from external sources							Priority status	Date for nomenclatural purposes
	Volume (year of the AAS meetings)	Date on title page of volume	Pages	Number of pages per volume	Signatures	The *Spraw. AU*		The *Rocznik AU*	Accession book at the Jagiellonian Library	Date of accession to Tromsø Museum	Gebethner's Catalogue	Köhler (2004)†		
						Bibliography	Meeting Report							
[1]	4B(44B) (1904)	1904	74–113	523	5–8	Aug.–Oct. 1905 (*Spraw. AU* 10(8): 2–3, 1905)	DD	1 May 1905–30 Apr. 1906 (*Rocznik AU* 1905–1906: 115–116)	DD	1906 (*Tromsø Mus. Aarsberetn.* 1906: 18. 1907)	Nov. 1905 (Świszczowski 1905: 13)‡	1904 [issued 1905]	original	Aug.–Oct. 1905
2			153–196		10–13									
3			305–341		20–22									
4	5B(45B) (1905)	1906	83–110	550	6–7	Aug.–Oct. 1906 (*Spraw. AU* 11(8): 3–4, 1906)	DD	1 May 1906–30 Apr. 1907 (*Rocznik AU* 1906–1907: 145–156)	19 Nov. 1906	1907 (*Tromsø Mus. Aarsberetn.* 1907: 20. 1908)	Dec. 1906 (Świszczowski 1906: 28)§	1906	simultaneously with Volume 1 of the book	Aug.–Oct. 1906
5	6B(46B) (1906)	1906	65–102	701	5–7	Mar. 1907 (*Spraw. AU* 12(3): 2–3, 1907)	DD	1 May 1906–30 Apr. 1907 (*Rocznik AU* 1906–1907: 145–156)	23 May 1907	1907 (*Tromsø Mus. Aarsberetn.* 1907: 20. 1908)	Dec. 1906 (in case of Part 7 only pp. 241–288) (Świszczowski 1906: 29)§	1906	reprint	Dec. 1906
6			189–239		12–15									
7			241–296		16–19									Dec. 1906 (pp. 241–288) / Mar. 1907 (pp. 289–296)
8	7B(47B) (1907)	1907	153–236	758	10–15	Mar. 1908 (*Spraw. AU* 13(3): 2, 1908)	DD	1 May 1907–30 Apr. 1908 (*Rocznik AU* 1907–1908: 145–146)	7 May 1908	DD	Jan.–Feb. 1908 (Świszczowski 1908: 42)‖	1907	original	Jan.–Feb. 1908
9			265–302		17–19									
10			587–631		37–40									
11			685–703		43–44						May–Jun. 1908 (Świszczowski 1908: 75)¶			Mar. 1908
12	8B(48B) (1908)	1909	41–90	529	3–6	Mar. 1909 (*Spraw. AU* 14(3): 2, 1909)	DD	1 May 1908–30 Apr. 1909 (*Rocznik AU* 1908–1909: 164–165)	DD	DD	Jul.–Aug. 1908 (Świszczowski 1908: 85–86)#	1908 [issued 1909]	original	Jul.–Aug. 1908
13			187–256		12–16									
14			331–336		21						Apr.–May 1909 (Świszczowski 1909: 61)††			Mar. 1909
15	10B(50B) (1910)	1911	173–211	732	11–14	Jan. 1911 (*Spraw. AU* 16(1): 1–2, 1911)	DD	1 May 1910–30 Apr. 1911 (*Rocznik AU* 1910–1911: 164–165)	DD	DD	Feb.–Mar. 1911 (Świszczowski 1911: 11)‡‡	1910 [issued 1911]	original	Jan. 1911
16			353–408		23–26									
17			671–682		42–43									
18	11B(51B) (1911)	1911	1–34	525	1–3	Mar. 1912 (*Spraw. AU* 17(3): 2, 1912)	4 Mar. 1912 (*Spraw. AU* 17(3): 18, 1912)	1 May 1911–30 Apr. 1912 (*Rocznik AU* 1911–1912: 165–166)	DD	DD	[not issued]	1911	reprint	Mar. 1912
19			67–89		5–6									
20			121–157		8–10									
21			265–280		17–18									
22			443–456		28–29								original	
23	12B(52B) (1912)	1912	1–49	565	1–4	Feb. 1913 (*Spraw. AU* 18(2): 2, 1913)	3 Mar. 1913 (*Spraw. AU* 18(3): 15, 1913)	1 May 1912–30 Apr. 1913 (*Rocznik AU* 1912–1913: 192–193)	DD	DD	[not issued]	1912	original	Feb. 1913
24			157–180		10–12									
25			211–239		14–15									
26			547–565		35–36									
27	13B(53B) (1913)	1913	29–49	619	2–4	Apr. 1914 (*Spraw. AU* 19(4): 2–3, 1914)	27 Apr. 1914 (*Spraw. AU* 19(4): 14, 1914)	1 May 1913–30 Apr. 1914 (*Rocznik AU* 1913–1914: 117–118)	DD	DD	[not issued]	1913	original	Apr. 1914
28			115–125		8									
29			311–339		20–22									
30	14B(1)(54B(1)) (1914)	1914	209–270	422	14–17	Oct.–Dec. 1914 (*Spraw. AU* 19(8–10): 3, 1914)	26 Oct. 1914 (*Spraw. AU* 19(8–10): 19, 1914)	1 May 1914–30 May 1916 (*Rocznik AU* 1914–1916: 126)	DD	DD	[not issued]	1914	original	Oct.–Dec. 1914

† Köhler P (2004) Bibliografia botaniki w Towarzystwie Naukowym Krakowskim, Akademii Umiejętności i Polskiej Akademii Umiejętności. – Bibliography of botany at the Academic Society of Cracow, Academy of Sciences and Letters, and the Polish Academy of Sciences and Letters (1818–1952–2000). W. Szafer Institute of Botany, Polish Academy of Sciences, Kraków.
‡ Świszczowski F (Ed.) (1905) Katalog Nowych Książek: miesięcznik bibliograficzny Księgarni G. Gebethnera i Spółki w Krakowie 2(1): 1−16. https://jbc.bj.uj.edu.pl/dlibra/publication/343922/edition/328470#structure
§ Świszczowski F (Ed.) (1906) Katalog Nowych Książek: miesięcznik bibliograficzny Księgarni G. Gebethnera i Spółki w Krakowie 3(2): 17−32. https://jbc.bj.uj.edu.pl/dlibra/publication/343922/edition/328470#structure
‖ Świszczowski F (Ed.) (1908) Katalog Nowych Książek: miesięcznik bibliograficzny Księgarni G. Gebethnera i Spółki w Krakowie 4(3-4): 29−44. https://jbc.bj.uj.edu.pl/dlibra/publication/343922/edition/328470#structure
¶ Świszczowski F (Ed.) (1908) Katalog Nowych Książek: miesięcznik bibliograficzny Księgarni G. Gebethnera i Spółki w Krakowie 4(7-8): 61−76. https://jbc.bj.uj.edu.pl/dlibra/publication/343922/edition/328470#structure
# Świszczowski F (Ed.) (1908) Katalog Nowych Książek. miesięcznik bibliograficzny Księgarni G. Gebethnera i Spółki w Krakowie 4(9-10): 77−88. https://jbc.bj.uj.edu.pl/dlibra/publication/343922/edition/328470#structure
†† Świszczowski F (Ed.) (1909) Katalog Nowych Książek: miesięcznik bibliograficzny Księgarni G. Gebethnera i Spółki w Krakowie 5(6-7): 57−72. https://jbc.bj.uj.edu.pl/dlibra/publication/343922/edition/328470#structure
‡‡ Świszczowski F (Ed.) (1911) Katalog Nowych Książek: miesięcznik bibliograficzny Księgarni G. Gebethnera i Spółki w Krakowie 8(2-3): 17−40. https://jbc.bj.uj.edu.pl/dlibra/publication/343922/edition/328470#structure
