# Supplementary material for: Zapałowicz’s Conspectus florae Galiciae criticus: Clarification of publication dates for nomenclatural purposes and bibliographic notes
Source: PhytoKeys. 2020 Aug 7;155:53–85. doi: 10.3897/phytokeys.155.51072 (PMC7443691; doi:10.3897/phytokeys.155.51072)
Supplement: Supplementary material 3 — Table S3 [file phytokeys-155-053-s003.pdf]

Supplementary material 3. Receipts for the Academy of Arts and Sciences provided by the Jagiellonian University Printing House for printing subsequent signatures of the *Rozprawy Wydziału Matematyczno-Przyrodniczego Akademii Umiejętności, Dział B. Nauki Biologiczne (Seria 3)* (the *Rozprawy*) preserved in the Archives of the Jagiellonian University. Abbreviations: DD – Data deficient.

<table>
<tr><th>Date</th><th>Receipt no.</th><th>Volume</th><th>Fascicle composition of the Rozprawy</th><th>Signatures printed and finishing activities</th><th>Reference code</th></tr>
<tr><td>30 Jun. 1904</td><td>5474</td><td rowspan="6">4B(44B)</td><td rowspan="6">DD</td><td>1–4</td><td>DUJ 185</td></tr>
<tr><td>30 Jun. 1904</td><td>5586</td><td>5–8</td><td>DUJ 185</td></tr>
<tr><td>9 Aug. 1904</td><td>5670</td><td>9–16</td><td>DUJ 185</td></tr>
<tr><td>29 Dec. 1904</td><td>5877</td><td>17–21</td><td>DUJ 185</td></tr>
<tr><td>1 Jun. 1905</td><td>248</td><td>22–31</td><td>DUJ 186</td></tr>
<tr><td>30 Dec. 1905</td><td>584</td><td>32–33, postpress operations for vol. 44B</td><td>DUJ 186</td></tr>
<tr><td>1 Jun. 1905</td><td>250</td><td rowspan="3">5B(45B)</td><td rowspan="3">DD</td><td>1–7</td><td>DUJ 186</td></tr>
<tr><td>5 Oct. 1905</td><td>438</td><td>8–20</td><td>DUJ 186</td></tr>
<tr><td>30 Dec. 1905</td><td>585</td><td>21–30</td><td>DUJ 186</td></tr>
<tr><td>1906</td><td>DD</td><td>DD</td><td>DD</td><td>DD</td><td>DD</td></tr>
<tr><td>29 May 1907</td><td>1589</td><td>6B(46B)</td><td>DD</td><td>Postpress operations for vol. 46B</td><td>DUJ 187</td></tr>
<tr><td>29 May 1907</td><td>1591</td><td rowspan="4">7B(47B)</td><td rowspan="4">Fascicle 1: 1–336 (signatures 1–21),
Fascicle 2: 337–640 (signatures 22–40),
Fascicle 3: 641–758 (signatures 41–48)</td><td>1–15</td><td>DUJ 187</td></tr>
<tr><td>3 Aug.1907</td><td>1725</td><td>16–26</td><td>DUJ 187</td></tr>
<tr><td>8 Oct. 1907</td><td>1852</td><td>27–40</td><td>DUJ 187</td></tr>
<tr><td>5 May 1908</td><td>2402</td><td>41–47, postpress operations for vol. 47B</td><td>DUJ 188</td></tr>
<tr><td>5 May 1908</td><td>2404</td><td rowspan="4">8B(48B)</td><td rowspan="4">Fascicle 1: 1–256 (signatures 1–16),
Fascicle 2: 257–405 (signatures 17–25),
Fascicle 3: 406–519 (signatures 26–33)</td><td>1–10</td><td>DUJ 188</td></tr>
<tr><td>7 Aug. 1908</td><td>2577</td><td>11–24</td><td>DUJ 188</td></tr>
<tr><td>22 Dec. 1908</td><td>2865</td><td>25–28</td><td>DUJ 188</td></tr>
<tr><td>15 May 1909</td><td>3145</td><td>29–33, 2/16 of 34, postpress operations for vol. 48B</td><td>DUJ 189</td></tr>
<tr><td>15 May 1909</td><td>3147</td><td rowspan="4">9B(49B)</td><td rowspan="4">DD</td><td>1–7</td><td>DUJ 189</td></tr>
<tr><td>30 Sep. 1909</td><td>3455</td><td>8–13</td><td>DUJ 189</td></tr>
<tr><td>22 Dec. 1909</td><td>3626</td><td>14–18</td><td>DUJ 189</td></tr>
<tr><td>10 May 1910</td><td>256</td><td>Postpress operations for vol. 49B</td><td>DUJ 190</td></tr>
<tr><td>10 May 1910</td><td>258</td><td rowspan="4">10B(50B)</td><td rowspan="4">DD</td><td>1–14</td><td>DUJ 190</td></tr>
<tr><td>1 Oct. 1910</td><td>519</td><td>15–42</td><td>DUJ 190</td></tr>
<tr><td>24 Dec. 1910</td><td>727</td><td>43–45</td><td>DUJ 190</td></tr>
<tr><td>3 May 1911</td><td>1037</td><td>Postpress operations for vol. 50B</td><td>DUJ 191</td></tr>
<tr><td>3 May 1911</td><td>1039</td><td rowspan="4">11B(51B)</td><td rowspan="4">DD</td><td>1–4</td><td>DUJ 191</td></tr>
<tr><td>30 Sep. 1911</td><td>1293</td><td>5–23</td><td>DUJ 191</td></tr>
<tr><td>27 Dec. 1911</td><td>1453</td><td>24–32</td><td>DUJ 191</td></tr>
<tr><td>4 May 1912</td><td>1709</td><td>33, postpress operations for vol. 51B</td><td>DUJ 192</td></tr>
<tr><td>4 May 1912</td><td>1711</td><td rowspan="3">12B(52B)</td><td rowspan="3">DD</td><td>1–2</td><td>DUJ 192</td></tr>
<tr><td>1 Oct. 1912</td><td>1987</td><td>3–13</td><td>DUJ 192</td></tr>
<tr><td>21 Dec. 1912</td><td>2155</td><td>14–24</td><td>DUJ 192</td></tr>
<tr><td>1913</td><td>DD</td><td>DD</td><td>DD</td><td>DD</td><td>DD</td></tr>
<tr><td>30 May 1914</td><td>212</td><td>13B(53B)</td><td>DD</td><td>35–39, postpress operations for vol. 53B</td><td>DUJ 193</td></tr>
<tr><td>30 May 1914</td><td>214</td><td rowspan="2">14B(54B(1))</td><td rowspan="2">DD</td><td>1–10</td><td>DUJ 193</td></tr>
<tr><td>3 Oct. 1914</td><td>393</td><td>11–27, postpress operations for vol. 54B(1)</td><td>DUJ 193</td></tr>
</table>
