# Supplementary material for: Zapałowicz’s Conspectus florae Galiciae criticus: Clarification of publication dates for nomenclatural purposes and bibliographic notes
Source: PhytoKeys. 2020 Aug 7;155:53–85. doi: 10.3897/phytokeys.155.51072 (PMC7443691; doi:10.3897/phytokeys.155.51072)
Supplement: Supplementary material 4 — Table S4 [file phytokeys-155-053-s004.pdf]

Supplementary material 4. Dates of publications of Zapałowicz's three-volume book *Conspectus florae Galiciae criticus – Krytyczny przegląd roślinności Galicyi*. Abbreviations: Add. – Addenda; Corr. – Corrigenda; Gebethner's Catalogue – *Katalog Nowych Książek: miesięcznik bibliograficzny Księgarni G. Gebethnera i Spółki w Krakowie* [= Catalogue of New Books: bibliographic monthly journal of G. Gebethner & Co. Publishing House in Kraków], the *Rozprawy – Rozprawy Wydziału Matematyczno-Przyrodniczego Akademii Umiejętności, Dział B. Nauki Biologiczne (Seria 3)*, the *Spraw. AU – Sprawozdania z Czynności i Posiedzeń Akademii Umiejętności w Krakowie* [= Reports on the AAS's Activities and Meetings (Kraków)].

**Vol.**	**Part**	**Pages**	**Date on title page of volume**	**Dates of publication from external sources**		**Priority status**	**Date for nomenclatural purposes**
				**The *Spraw. AU*: Bibliography section**	**Gebethner's Catalogue**		
1(†)	[1]	1–40	1906	Aug.–Oct. 1906 (*Spraw. AU* 11(8): 3−4, 1906)	Oct. 1906 (Świszczowski 1906: 118)‡	reprint	Aug.–Oct. 1906
	2	40–83				reprint	
	3	83–119				reprint	
	4	119−146				simultaneously with Vol. 48B of the *Rozprawy*	
	5	146−183				original	
	6	184−234				original	
	7	234−289				original	
	Add. to Vol. 1	291−294				original	
	Index generum	295−296					
	Corr. for Vol. 1	[297]				original	
2	8	1−84	1908	Aug.–Oct. 1908 (*Spraw. AU* 13(8): 2, 1908)	Sept.–Oct. 1908 (Świszczowski 1908: 102)§	reprint	Aug.–Oct. 1908
	9	84−121				reprint	
	10	121−164				reprint	
	11	164−183				reprint	
	12	183−231				reprint	
	13	231−300				reprint	
	Add. to Vols 1, 2	301−311				original	
	Corr. for Vols 1, 2	[313−314]				original	
	Index generum	[315]					
3	14	1−6	1911	Nov. 1911 (*Spraw. AU* 16(9): 1, 1911)	Jan.–Febr. 1912 (Świszczowski 1912: 14)|	reprint	Nov. 1911
	15	6−44				reprint	
	16	44−98				reprint	
	17	98−109				reprint	
	18	109−142				original	
	19	142−164				original	
	20	164−200				original	
	21	200−216				original	
	Index generum	[217]					
	Corr. for Vols 1, 2, 3	219				original	
	Add. to Vols 1, 2, 3	221−246				original	

† 19 Nov. 1906 − receipt date in the receipt book of the Jagiellonian Library

‡ Świszczowski F (Ed.) (1906) Katalog Nowych Książek: miesięcznik bibliograficzny Księgarni G. Gebethnera i Spółki w Krakowie 2(12): 113−120. https://jbc.bj.uj.edu.pl/dlibra/publication/343922/edition/328470#structure

§ Świszczowski F (Ed.) (1908) Katalog Nowych Książek: miesięcznik bibliograficzny Księgarni G. Gebethnera i Spółki w Krakowie 4(11–12): 89−104. https://jbc.bj.uj.edu.pl/dlibra/publication/343922/edition/328470#structure

| Świszczowski F (Ed.) (1912) Katalog Nowych Książek: miesięcznik bibliograficzny Księgarni G. Gebethnera i Spółki w Krakowie 9(1–2): 1–20. https://jbc.bj.uj.edu.pl/dlibra/publication/343922/edition/328470#structure
